# Perinatal Maturation of Drug Transporters and Claudin‐5 at the Blood–Brain Barrier

**DOI:** 10.1111/cns.70614

**Published:** 2025-09-18

**Authors:** Laetitia Federici, Salvatore Cisternino, Sylvain Auvity, Antoinette Gelot, Mathilde Becmeur‐Lefebvre, Maryline Favier, Gaelle Letort, Philippe Mailly, Martine Cohen‐Salmon, Anne‐Cécile Boulay

**Affiliations:** ^1^ Université Paris Cité, INSERM, Optimisation thérapeutique en neuropharmacologie OTEN U1144 Paris France; ^2^ Service Pharmacie, Assistance Publique – Hôpitaux de Paris, AP‐HP, Hôpital Universitaire Necker – Enfants Malades Paris France; ^3^ Service d'anatomie et cytologie pathologie, AP‐HP, Hôpital Armand Trousseau Paris France; ^4^ Service de foetopathologie Centre hospitalier régional d'Orleans Orléans France; ^5^ HistIM Facility Institut Cochin Paris France; ^6^ Center for Interdisciplinary Research in Biology (CIRB), Collège de France, CNRS, INSERM, Université PSL Paris France

**Keywords:** blood–brain barrier, development, drug transporters, endothelial cell, Oatp1a4, P‐glycoprotein, rosuvastatin, verapamil

## Abstract

**Aim:**

Cerebral capillary endothelial cells (EC) form the blood–brain barrier (BBB), which regulates molecular exchange between the blood and the brain. Understanding their function during brain development is essential for optimizing treatments in neonates, children, as well as pregnant and breastfeeding women.

**Methods:**

P‐glycoprotein (P‐gp/ABCB1) expression during brain development was assessed by immunohistochemistry in human cortical samples. In mice, postnatal brain microvessels were analyzed using qPCR and Western Blot, and BBB function was evaluated in vivo using [^14^C]sucrose to assess barrier integrity, and [^3^H]verapamil or [^3^H]rosuvastatin to assess transport activity.

**Results:**

In humans, P‐gp reached mature levels in the early postnatal period. In mice, BBB integrity was established by postnatal day 5 (P5), but the expression of claudin‐5, P‐gp, and Oatp1a4 increased until P30. Brain transport of verapamil and rosuvastatin significantly decreased between P15 and P30, indicating enhanced efflux capacity.

**Conclusions:**

Although BBB integrity is established at birth, BBB continues maturing throughout the postnatal period, with a predominant efflux transport. Our findings underscore the critical role of P‐gp in the acquisition of BBB gatekeeper properties. The immature BBB may result in a higher brain susceptibility to P‐gp substrates in preterm infants.

## Graphical abstract

P‐gp expression in the developping human cortex increases with age. In the mouse, gatekeepers of BBB integrity and transport are upregulated: tighter blood–brain barrier, increased claudin 5/Slco1a4/P‐gp expression as well as enhanced P‐gp‐mediated BBB efflux.

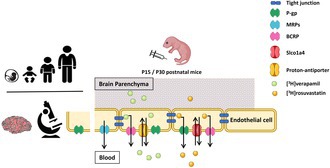

## 1 Introduction

Pharmacokinetics (PK) allows us to determine how molecules and, in particular, drugs are distributed within an organism. A better understanding of how PK evolves with age is essential for more effective and safer use of therapies adapted to each age. This is particularly crucial in the treatment of the fetus, premature infants, and children. In the early stages of fetal development, two biological interfaces designed as barriers are of critical importance for PK: the placenta and the blood–brain barrier (BBB). The placental barrier allows a controlled molecular exchange between the maternal and fetal circulations, providing essential nutrients and protecting the fetus from toxins and pathogens [1]. The BBB also acts as a selective barrier to regulate exchanges between the fetal blood and brain parenchyma.

Much is known about the specific features of the brain endothelium that contribute to BBB properties, distinguishing it from the endothelium of peripheral tissues [2]. During brain development, BBB ECs undergo a series of molecular and histological changes to meet the needs of the growing brain while maintaining its protection and homeostasis. How this dual role is achieved during late embryonic and postnatal development remains, however, unclear.

The paracellular passage between brain ECs of the BBB is highly restricted by multiprotein complexes forming adherens and tight junctions (TJ). Claudin‐5 is a critical component of these TJ complexes [3] along with Occludin and ZO‐1 [4]. In mice, TJ formation is initiated during embryonic development but may continue to mature during postnatal development [5].

Because of the presence of TJs between ECs, a complex substrate‐specific control of the exchange is set at the BBB mediated by two major membrane transporter superfamilies: ATP‐binding cassette (ABC) and solute carrier (SLC) transporters [6]. At the luminal membrane, ABC transporters such as P‐glycoprotein (P‐gp, ABCB1, *Abcb1a*) participate in the unidirectional efflux of substrates from the endothelial cell into the blood, thus limiting exposure of the brain to neurotoxic compounds and numerous drugs. Other ABC transporters such as BCRP (ABCG2) or MRP (e.g., MRP4/ABCC4) are also involved in restricting transcellular exchange at the BBB, using shared or specific substrate recognition [7]. In contrast to the ABC, SLC transporters allow the influx into the brain of a wide range of endogenous compounds (e.g., glucose, amino acids, monocarboxylates) and a variety of drugs. The functional characteristics and competence of the immature and term brain endothelium remain to be further explored in the pediatric population to better guide therapeutic approaches. Previous molecular studies have shown rather complex expression changes including up‐ or down‐regulation in ABC and SLC transporters [8, 9]. Changes in the activity of transporters involved in drug transport may therefore cause PK variability in efficacy and toxicity.

In this study, we explored the expression of P‐gp in human tissue from perinatal periods to 17 years old. In parallel, we analyzed in mice the expression of claudin‐5, P‐gp, and Slco1a4 between P5 and P60. For a better understanding of the complexity of the transport interplay, we combined this molecular analysis with a functional assessment of drug brain distribution in young mice using mixed ABC/SLC substrate probes: verapamil, a dual P‐gp and organic cation/proton‐antiporter substrate, and rosuvastatin, a known P‐gp/BCRP/Slco1a4 substrate. We observed a marked increase in both the expression and functionality of efflux transporters during the postnatal maturation of the mouse brain, as well as a perinatal rise in P‐gp expression in humans.

## 2 Material and Methods

### 2.1 P‐gp Expression Quantification on Human Tissue Samples

The specimens described here are part of the “Hôpitaux Universitaires de l'Est Parisien – Neuropathologie du développement” brain collection (biobank identification number: BB‐0033‐00082). Informed consent was obtained for brain autopsy and histological examination. Fetal brains were obtained from spontaneous or medical abortions. The fetuses did not display any significant brain disorders or diseases. Analyzed samples were from prenatal stages (15 week of gestation [wg]; 21 wg; 28 wg; 30 wg; 39 wg) and postnatal stages (3 weeks; 1 month; 2 months; 3 months; 8 months; 1 year; 3 years; 4 years [*n* = 2]; 10 years; 11 years; 12 years; 13 years [*n* = 2]; 16 years; 17 years). One slice per sample was analyzed.

The same technical procedures were applied to all brain samples: after removal, brains were fixed with formalin for 5–12 weeks. A macroscopic analysis enabled the samples to be selected and processed (paraffin embedding, preparation of 7 μm slices, and staining with hematein reagent) for histological analysis. Coronal slices including the temporal telencephalic parenchyma and the hippocampus were deparaffinized and unmasked in citrate buffer (pH 6.0). Expression of P‐gp was detected using the Bond Polymer Refine Detection kit (Leica) and processed on automated Bond RX Leica immunostaining systems (Table S1). Pictures were acquired using a slide scanner (Lamina, Perkin Elmer).

Stained samples were analyzed using the QuPath software [10] (Table S1). For each sample, a QuPath “pixel classifier” was trained to discriminate between 3,3′‐diaminobenzidine (DAB)‐positive spots and the background. This “classifier” consisted of an artificial neural network based on four features: a Gaussian filter to select for the intensity, and three structure tensor eigenvalues to favor thin elongated objects. To train the classifier, we defined manually annotated spots and background areas on one image per developmental stage. When the results were visually satisfactory, the trained pixel classifier was used to detect positive spots in manually defined regions of interest.

### 2.2 Experiments on Mice and Ethical Approval

Swiss mice were purchased from Janvier Labs (Le Genest‐Saint‐Isle, France) and kept in pathogen‐free conditions. All animal experiments were carried out in compliance with (i) the European Directive 2010/63/EU on the protection of animals used for scientific purposes and (ii) the guidelines issued by the French National Animal Care and Use Committee (reference: 2013/118). The study was also approved by the French Ministry for Research and Higher Education's institutional review board. Males and females were equally included in all experiments.

### 2.3 Molecular Experiments in Mice

#### 2.3.1 Brain Microvessels Purification

Microvessels (MV) were purified from whole brains, as described previously [11]. A 100 μm‐mesh negative filter and a 20 μm‐mesh positive filter were used to enrich our preparation with arterioles, venules, and capillaries, and remove cell debris. This preparation allows the purification of the total neurogliovascular unit, which comprises not only the vascular cells (ECs, mural cells, fibroblasts, perivascular macrophages) but also attached astrocyte endfeet and neuronal fibers with their mRNAs and proteins content [12]. The purification process does not change the cellular composition of the MVs at any stage, as previously shown [13].

#### 2.3.2 Quantitative RT‐PCR

Total RNAs were extracted using the RNeasy Mini Kit (Qiagen). cDNAs were generated from 100 ng of RNA using the Superscript III Reverse Transcriptase Kit. Differential levels of cDNA expression were measured using droplet digital PCR (ddPCR). Briefly, cDNA and primers were distributed into approximately 10,000–20,000 droplets. cDNAs were then PCR‐amplified in a thermal cycler and read (as the number of positive and negative droplets) on a QX200 ddPCR system (Biorad, Hercules, CA, USA). The ratio for each tested gene was normalized against the total number of positive droplets for *Gapdh*. The primer sequences are given in Table S1. Three to five independent samples were analyzed.

#### 2.3.3 Western Blots

MV pellets were sonicated three times for 10 s at 20 Hz (Vibra cell VCX130) in 2% SDS and heated at 56°C in Laemmli loading buffer (Biorad). The protein content was measured using the Pierce 660 nm protein assay kit (Thermo Scientific, Waltham, MA, USA). 10 μg of proteins were separated by denaturing electrophoresis on a 4%–15% Criterion TGX Precast Midi Protein Gel (Biorad) and then electro‐transferred to nitrocellulose membranes using the Trans‐Blot Turbo Transfer System (Biorad). Membranes were hybridized, as described previously [14]. The antibodies used in the present study are listed in Table S1. Horseradish peroxidase activity was visualized by enhanced chemiluminescence in a Western Lightning Plus system (Perkin Elmer, Waltham, MA, USA). Chemiluminescent imaging was performed on a *FUSION FX* system (Vilber, South Korea). The level of chemiluminescence for each antibody was normalized against the histone‐3 staining on the membrane.

### 2.4 Functional Experiments in Mice

#### 2.4.1 Radiolabeled Compounds

[^14^C]sucrose (specific activity [SA] 20.1 GBq/mmol), and [^3^H]verapamil (SA 304.9 GBq/mmol) were purchased from Perkin Elmer (Paris, France). [^3^H]rosuvastatin (SA 203.5 GBq/mmol) was purchased by Moravek Inc. (Brea, CA, USA).

#### 2.4.2 Experimental Procedure

Mice were i.p. injected with saline (10 mL/kg) containing the [^3^H]verapamil (~0.074 MBq/mL) or [^3^H]rosuvastatin (~0.130 MBq/mL) and the vascular marker [^14^C]sucrose (~0.031 MBq/mL). Mice were then decapitated according to selected time points, with blood samples collected following decapitation. The brain was then removed from the skull. For each mouse, the brain hemispheres and two blood samples were weighed in Econo glass vials for liquid scintillation counting (Perkin Elmer). All samples were treated with a tissue solubilizer (Solvable, Perkin Elmer). Blood samples were in addition decolored with 0.2 mL of 30% hydrogen peroxide solution to avoid color quenching issues. After complete tissue sample digestion (24 h in a water bath at 45°C), the vials were cooled and mixed with Ultima‐gold XR (Perkin Elmer). The radioactivity in each vial, expressed in disintegrations per minute (dpm) for ^3^H and ^14^C, was counted in a Perkin Elmer Tri‐Carb counter.

For [^3^H]verapamil, the experimental procedure was performed at 2.5 and 5 min post i.p. [^3^H]verapamil injection (7 and 6 mice at P15 respectively; 7 and 6 mice at P30 respectively). For [^3^H]rosuvastatin, data collection was performed at 5 and 7 min post i.p. injection. The elimination half‐life of these compounds is much longer than the times chosen for the experiments. This avoids quantification bias caused by the formation of possible radiolabelled metabolites [15].

#### 2.4.3 Data Quantification

Brain uptake of [^3^H]verapamil and [^3^H]rosuvastatin was determined using the Patlak plot analysis [16] from 0 to the time points studied. First, the vascular volume (*V*_vasc_, μL/g) was estimated for each mouse as the brain volume of distribution (*V*_d_) of [^14^C]sucrose:

[^14^C]sucrose *V*_d_ was calculated as the ratio of midbrain to blood carbon‐14 associated radioactivity (dpm/g) measured at 5 min post i.p. injection.

$$V_{\text{vasc}}=V_{d_{\text{sucrose}}}=\frac{X_{\text{sucrose},\text{brain}}}{C_{\text{sucrose},\text{blood}}}$$

Where *X*_sucrose,brain_ (dpm/g), is the calculated amount of [^14^C]sucrose from the brain and *C*_sucrose,blood_ (dpm/μL) its blood concentration. The data for any animal whose *V*_vasc_ was an aberrant value was excluded from the study.

Then, the apparent brain distribution (μL/g) for [^3^H]verapamil or [^3^H]rosuvastatin was calculated as:

$$V_{b_{\text{compund}}}=\frac{X_{\text{compound},\text{brain}}}{C_{\text{compound},\text{blood}}}$$

Where *X*_compound,brain_ (dpm/g), is the calculated amount of [^3^H]compound from the brain and *C*_compound,blood_ (dpm/μL) its blood concentration. This total activity was corrected for “vascular” contamination using the *V*_vasc_ subtraction:

$$\begin{array}{c} X_{\text{compound},\text{brain}}=X_{\text{total},\text{brain}}-V_{\text{vasc}}\times C_{\text{compound},\text{blood}} \end{array}$$

Where *X*_total,brain_ (dpm/g), is the total quantity of [^3^H]compound measured in the brain tissue sample.

The brain uptake clearance for each [^3^H]compound (*K*_in_, μL/min/g) was determined by applying the following equation:

$$K_{\text{in}}=\frac{V_{b_{\text{compound}}}\times C_{\text{compound},\text{blood}}}{{\mathrm{AUC}}_{\text{compound},\text{blood}}}$$

Where AUC_compound,blood_ (dpm.min/μL) is the area under the blood concentration time curve of the [^3^H]compound from 2.5 to 5 min and from 5 to 7 min for [^3^H]verapamil and [^3^H]rosuvastatin, respectively.

### 2.5 BBB Integrity in Mice

The vascular volume based [^14^C]sucrose calculation was used as the outcome parameter for BBB integrity. Higher levels in *V*_vasc_ indicate leakage of [^14^C]sucrose into the brain parenchyma because of BBB leakage.

### 2.6 Statistics

Normal distribution of the data was assessed using the Kolmogorov–Smirnov test. For Western Blot and ddPCR analysis, the number of data points was too small. We thus performed non‐parametric analyses (Kruskal–Wallis test [overall, in bold] and one‐tailed Mann–Whitney test [comparison of stages] for 3–5 samples of MV per stage). For transport experiments, part of the data was not normally distributed; thus, we used exclusively non‐parametric tests (Kruskal–Wallis and Mann–Whitney tests). Statistical significance was set at *p* < 0.05.

## 3 Results

### 3.1 Developmental Expression Profile of P‐gp in the Human Cortex

**FIGURE 1:**
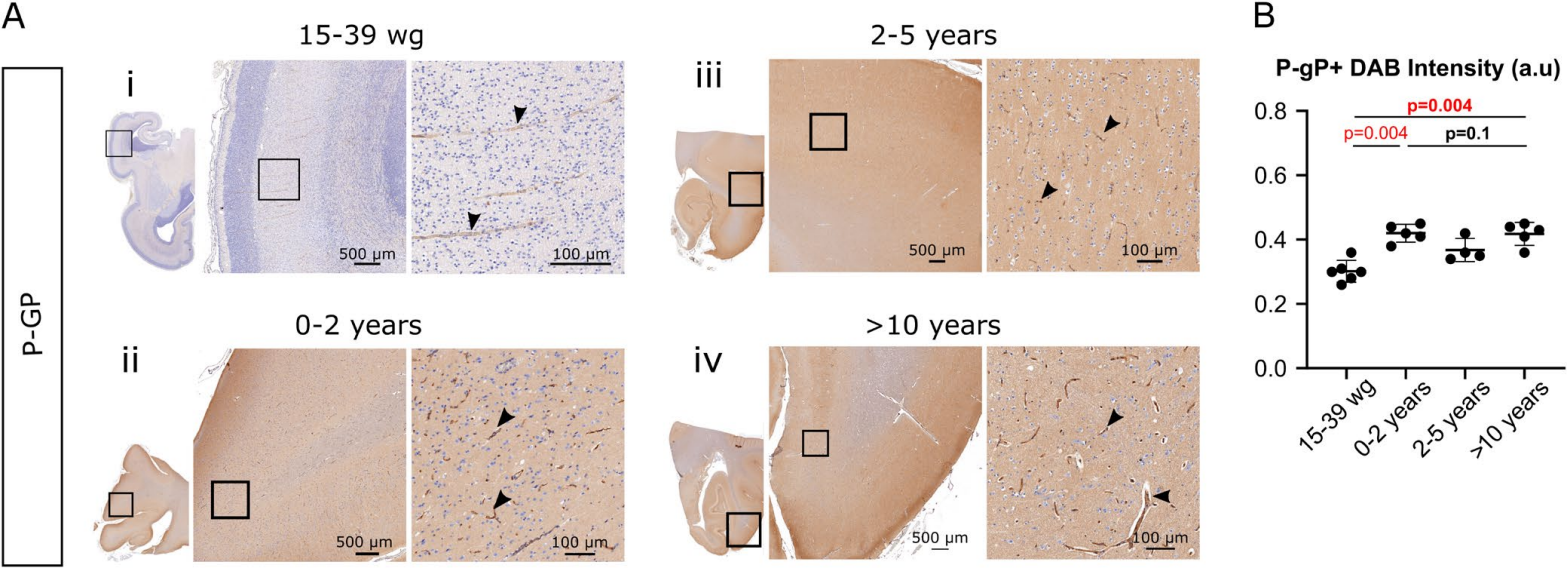
P‐gp expression in the developing human cortex. (A) Representative images of P‐gp‐immunostained cortical slices (left) and (at higher magnification) in the parenchyma of the boxed areas (right) for samples taken (i) in the prenatal period (ii), at 0–2 years of age, (iii) between 2 and 5 years of age, and (iv) after 10 years of age. P‐gp immunostaining (arrowheads) was revealed by DAB staining. (B) DAB intensity was quantified and quoted as the mean ± SD.

We performed an immunohistochemical analysis of P‐gp in non‐diseased human cortical sections from 15 weeks of gestation (wg) to 17 years of age (Figure 1). P‐gp was already present in the brain vessels at 15 wg (Figure 1A, Table S2). The level of P‐gp in brain vessels increased at birth and then stabilized (Figure 1B, Table S2).

### 3.2 Postnatal Maturation of Endothelial BBB Molecular Properties in Mice

**FIGURE 2:**
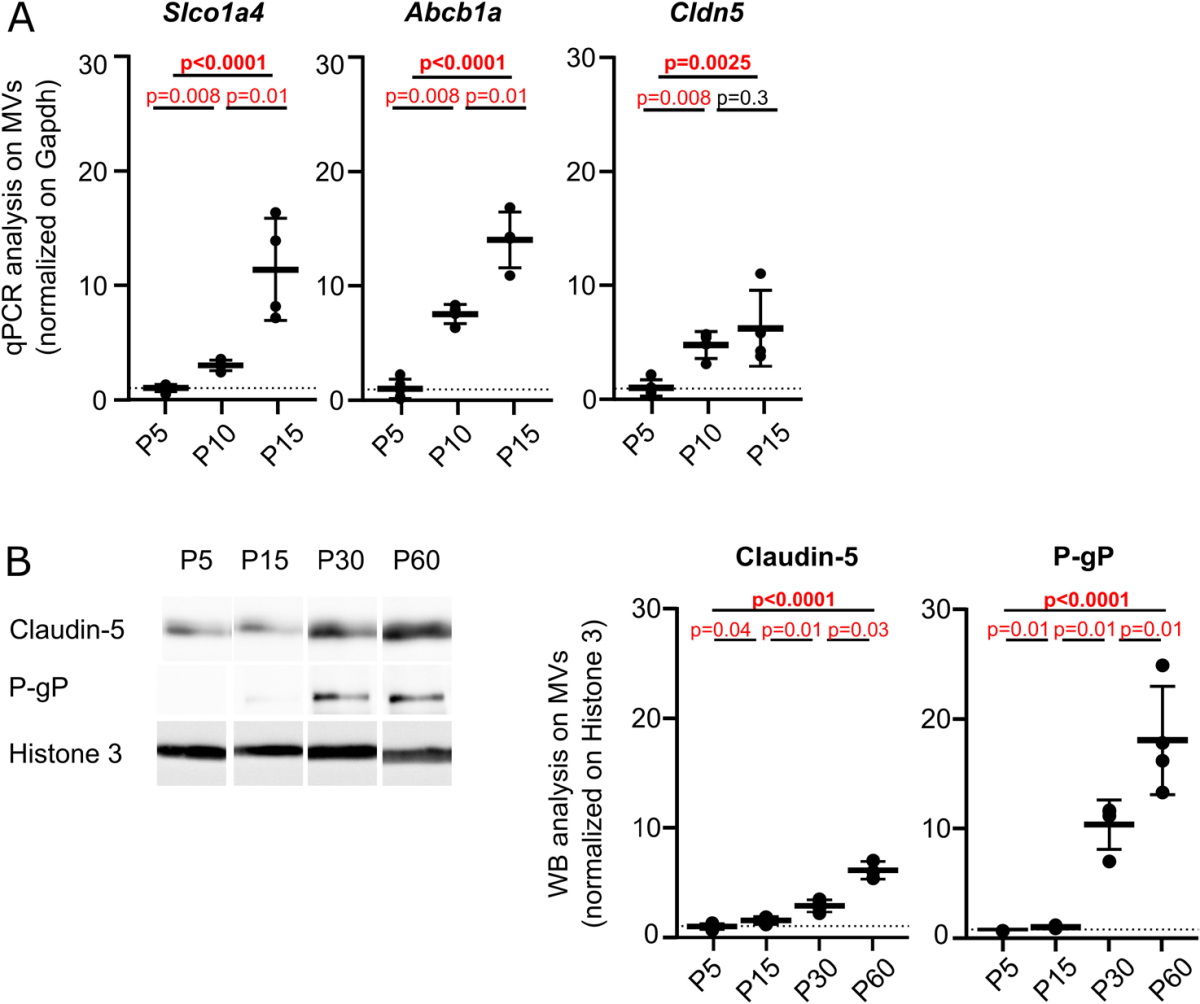
Postnatal molecular maturation of endothelial BBB components in mice. (A) mRNA analysis for *Cldn‐5*, *Abcb1a* and *Slco1a4* at P5, P10 and P15 in purified MVs from whole brains. (B) Protein quantification of claudin‐5 and P‐gp in purified MVs from whole brains at P5, P15, P30 and P60. Signals were normalized on Histone H3. P5 value is set as 1. Kruskal–Wallis test (overall, in bold) and a one‐tailed Mann–Whitney test (comparison of stages). The data are quoted as the mean ± SD (*n* = 3 or 4 samples per developmental stage; mice per sample: 5 for P5; 4 for P10; 3 for P15; 2 for P30 and P60).

To more broadly characterize BBB during brain development, we analyzed its maturation in the mouse cortex. Although the overall sequence of brain development is similar in mice and humans [17], the formation of the gliovascular unit and its maturation occurs postnatally in rodents. To study endothelial maturation, we re‐analyzed a previously published cortical microvessels (MVs) RNASeq dataset at Postnatal day (P) 5 and P15 [13]. Transcripts encoding known *adherens* or tight junction proteins specific to ECs (e.g., *Ocln*, *Pecam‐1*, *Cdh5*, *Cldn5, Jam2*) were expressed and remained unchanged between P5 and P15 (−1 < Log2Fc < 1 and/or *p*adj > 0.05) (Table S3). Among the 230 solute carrier (Slc) family transporters expressed, 11 were specific to ECs (Table S3). Notably, *Slco1a4* (encoding Oatp1a4) and *Slc2a1* (encoding Glut‐1) which are enriched in ECs, were upregulated (Log2Fc > 1, *p*adj ≤ 0.05) (Table S3) [13]. Of the 30 ATP‐binding cassette (ABC) transporter transcripts expressed in purified MVs, only the endothelial cell‐specific *Abcb1a*, encoding P‐glycoprotein (P‐gp), was significantly upregulated at P15 (Log_2_Fc = 1.22, *p*adj = 7.19.10^−15^) (Table S3). To confirm these results and further characterize the molecular changes in brain ECs, we performed qPCR analysis on selected EC‐specific BBB genes in whole brain‐purified MVs from P5, P10, and P15 mice. Analyzed genes included *Cldn5* (encoding the tight junction protein claudin‐5), *Abcb1a*, and *Slco1a4*. The results showed significant upregulation for the three transcripts between P5 and P15 (Figure 2A, Table S2). Expression changes were then assessed at the protein level on Western blots of purified MVs from P5, P15, P30, and P60 whole brain (except for Oatp1a4 for which no antibodies are available) (Figure 2B, Table S2). The amount of claudin‐5 increased progressively from P5 to P60 (Figure 2B, Table S2). Albeit with a time lag compared to mRNAs, the P‐gp protein level was higher from P30 onwards with a significant shift between P15 and P30 (Figure 2B, Table S2).

### 3.3 BBB Integrity and Transport Capacity

**FIGURE 3:**
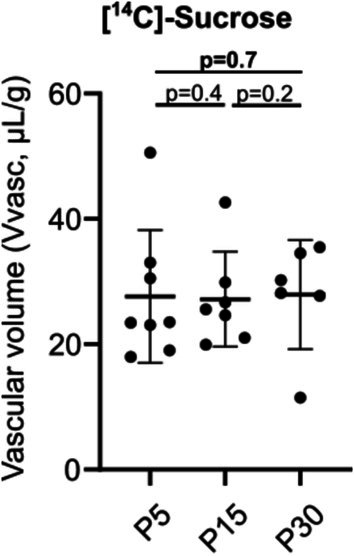
In vivo evaluation of BBB integrity in mice. Vascular volume of [^14^C]sucrose in mice at P5, P15 and P30. Data are represented as mean ± SD.

We next compared BBB integrity in vivo at P5, P15, and P30 by measuring the volume of distribution expressed as an apparent volume *V*_vasc_ (μL/g) of [^14^C]sucrose, a known marker of the vascular space and BBB physical integrity [18]. The [^14^C]sucrose *V*_vasc_ reached known physiological values and did not differ between P5, P15, and P30 (Figure 3, Table S2).

**FIGURE 4:**
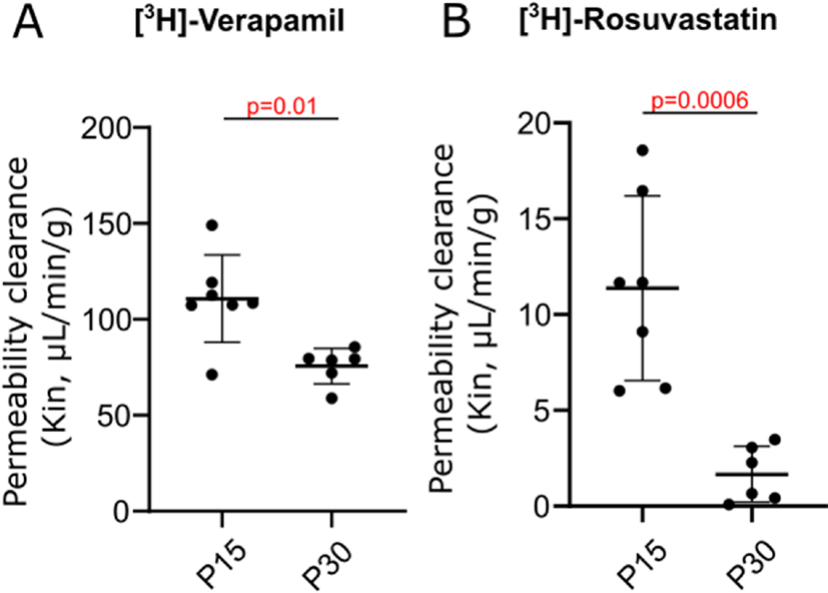
Postnatal maturation of permeability clearance of [^3^H]verapamil (A) and [^3^H]rosuvastatin (B) at the mice BBB. Data are represented as mean ± SD.

To evaluate in vivo the function of P‐gp, [^3^H]verapamil was injected in P15 or P30 mice (Figure 4A, Table S2). [^3^H]Verapamil cerebral transport decreased significantly by 1.6‐fold at P30 compared to P15. [^3^H]Rosuvastatin brain transport decreased significantly by 6.8‐fold at P30 compared to P15 (Figure 4B, Table S2).

## 4 Discussion

Information on pre‐ and postnatal vascular maturation from a molecular and functional perspective is scarce and controversial. TJs are important components of the BBB, providing a mechanical connection among ECs to maintain vascular structural integrity and prevent solute paracellular permeability [19]. In mice, we found an upregulation between P5 and P15 of claudin‐5, a major structural component of endothelial TJs. This suggests that, while set before birth, the BBB continues to mature postnatally. Several molecular and functional studies have shown that the mouse brain vasculature starts to develop around embryonic day (E)9 and that the brain endothelium tightly restricts the passage of molecules by E15 [20, 21]. BBB formation has been closely linked to the recruitment of brain pericytes during embryogenesis and measured by vascular markers such as 3 and 70 kDa dextran or albumin (67 kDa) [5, 22, 23, 24]. Here, we addressed BBB permeability to sucrose, a small size/molecular weight compound (342 g/mol). We found no change in brain accumulation between P5 and P30, suggesting that the paracellular permeability between the blood and the brain is restricted at P5 and onward. These results show that the “physical” barrier is established in newborn mice and that intermediate levels of claudin‐5 are sufficient in basal/physiological conditions [25]. In humans, intravenous injection of trypan blue in 10–31 wg fetuses shortly after cesarean delivery showed that the brain endothelium restricts paracellular permeability already in development [26]. In a recent single cell analysis of human fetal and adult brains, *CLDN5* was found expressed at a low level in ECs around 15 wg [27]. Immunohistochemistry and Western blot analyses also showed claudin‐5 expression at 16 wg [4, 28]. Thus, as in mice, TJ expression by ECs in humans is an early event which provides a first protection of the brain.

In addition to TJs, controlling the molecular exchanges between the blood and the brain requires specific ABC and SLC endothelial transporters. P‐gp is a major ABC transporter responsible for the BBB efflux and protection of the adult brain from a variety of drugs [29]. In mice, we noted a delayed increase in P‐gp expression following birth, occurring between P5 and P15/30. Ek et al. [30] also observed an increase in P‐gp between E13 and P7, and a stabilization on P21 [30]. In a human single cell study, fetal brain ECs showed very low *ABCB1* expression [27]. At the protein level, a weak P‐gp immunoreactivity was detected between 8 and 12 wg and 22 wg, with a gradual increase from 3 to 6 months of age to adult [31]. Our observations, which showed P‐gp expression at 15 wg and a perinatal increase, are therefore consistent with previous results.

Drug efflux at the BBB is also dependent on BCRP, whose substrates significantly overlap with P‐gp's. *Abcg2*, encoding BCRP, was unchanged between P5 and P15 in mouse MVs [13] and rats [30]. In contrast, *ABCG2* transcripts were found at a low level in fetal brains compared to adult stages [27]. However, brain immunohistochemical analysis of BCRP in human embryos and fetuses at 5–21 wg showed that BCRP expression was already mature at early stages [32]. Altogether, these results showed that the expression of ABC transporters varies across brain development. Immaturity at birth, at least for P‐gp‐mediated efflux, might put preterm infants more at risk for pharmaco‐toxicity when using therapy with P‐gp substrate drugs. This effect might be partially compensated by BCRP for dual P‐gp/BCRP substrates [33]. More investigation would therefore be necessary to identify the respective roles of these two ABC transporters in fetal/perinatal stages.

SLCs represent a large superfamily of transporters, essential for the influx of nutrients into the brain as well as drugs [34]. Diverse developmental profiles of SLC transporters have been described at the rodent BBB [35]. A recent transcriptomic analysis of purified mouse brain MVs showed that the level of *Slc2a1* and *Slco1a4* increased between P5 and P15 [13], confirmed here for *Slco1a4*. This is in line with earlier studies [36, 37, 38]. Oatp1a4, encoded by *Slco1a4*, is the mouse ortholog of OATP1A2/*OATP1A2*, and allows the influx of endogenous molecules (e.g., steroids) and drugs (e.g., statins, β‐blockers). Similar to results found in the mouse, *OATP1A2* transcripts were at a low level in human fetal brains compared to adult stages [27].

Expression of SLC and ABC transporters increases during brain development in both mice and humans. Understanding how these transporters functionally balance each other at the BBB during development is of high importance for pediatric care. Determining the quantitative contribution of a single transporter to the kinetics of substrates with multiple transporters is complex, even more so when opposing kinetic components coexist (e.g., ABC efflux and SLC influx). This complex interplay has been demonstrated and shows unpredictable substrate kinetics (i.e., additive, synergistic, concealed) [6]. In adult mice, the P‐gp‐mediated efflux of [^3^H]verapamil at the BBB has been shown to dominate and mask proton‐antiporter‐mediated influx, highlighting the critical role of P‐gp for this drug [39]. In the case of [^3^H]rosuvastatin, transport across BBB involves a combination of P‐gp, Bcrp, and Oatp1a4 [40, 41].

To determine how this influx/efflux interplay governs the access to the developing brain, we assessed drug transport into the mouse brain at P30 vs. P15. We observed decreased brain accumulation for both [^3^H]verapamil and [^3^H]rosuvastatin, suggesting a global increase in efflux capacity across the BBB, likely due to the observed increase in P‐gp expression. Likewise, Goralski et al. [42] reported a reduced brain/plasma ratio for two other P‐gp substrates, cyclosporine and digoxin, in P1 mice compared to adults; these differences were abolished in P‐gp null mice [42]. In rhesus monkeys, Takashima et al. [43] observed decreased BBB transport of [^11^C]verapamil in infants (9 months) compared to adolescents (2 years) and adults (4 years) using PET imaging [43]. In our study, [^3^H]verapamil was 1.6‐fold less transported at P30 than at P15, whereas [^3^H]rosuvastatin transport decreased 6.8‐fold. This greater reduction for [^3^H]rosuvastatin may reflect differences in transport capacity, possibly due to varying contributions of P‐gp.

Fetal protection is also provided by the placental barrier, where the expression of P‐gp has been shown to decrease from 6 wg to birth in humans and in a similar manner in the placentas of rodents [44], while BCRP expression does not change in human placentas at different gestational ages [44, 45, 46]. Combined with the observed immature fetal brain P‐gp expression, reduced placental P‐gp expression may increase the risk of a higher brain exposure to its substrate in late embryonic stages, and possibly more if it is not a dual P‐gp/BCRP substrate. Neonates and infants born at or before term are also at higher risk of developing undesirable pharmaco‐toxicological CNS effects [47] because, as shown, P‐gp is suboptimally expressed. As they may be subject to a wide range of pathologies and life‐threatening conditions, some of which may require the use of intensive care drugs (e.g., opiates, cardiotonic amines), particular attention should be paid to adverse effects on the CNS in term or preterm babies [47, 48].

In this study, we demonstrated that brain ECs follow postnatal molecular and functional maturation programs with the acquisition of specific properties which may impact drug BBB transport. Collectively, these data suggest a specialization of BBB transport systems during development. This may reflect changing needs of the brain in terms of maturation, nutrition, and detoxification, as evidenced by an increase in P‐gp efflux capacity. In order to understand and better predict the PK and pharmacotoxicological effects of the BBB on drug transport in the brain during these life stages, more quantitative information on the drug transport capacity (e.g., Km, Vmax, efflux ratio) toward P‐gp and drug transporters in general should be provided.

## Author Contributions

M.C.‐S., A.‐C.B., and S.C. designed research; L.F., S.C., M.B.‐L., M.F., G.L., M.C.‐S., and A.‐C.B. performed research; L.F., S.C., M.C.‐S., and A.‐C.B. analyzed data; S.A. and A.G. provided resources; L.F., S.C., M.C.‐S., and A.‐C.B. wrote the paper.

## Conflicts of Interest

The authors declare no conflicts of interest.

## Supporting information


**Table S1:** List of reagents and tools used in this article.
**Table S2:** List of data and results of statistical tests used in this article.
**Table S3:** Selection of transcript expression in RNASeq from cortical MV extracted between P5 and P15 (Slaoui et al. 2023).

## Data Availability

The data that support the findings of this study are available from the corresponding author upon reasonable request.
