# Supplementary material for: Perinatal Maturation of Drug Transporters and Claudin‐5 at the Blood–Brain Barrier
Source: CNS Neurosci Ther. 2025 Sep 18;31(9):e70614. doi: 10.1111/cns.70614 (PMC12446565; doi:10.1111/cns.70614)
Supplement: Supplementary file 1 — Table S1: List of reagents and tools used in this article. Table S2: List of data and results of statistical tests used in this article. Table S3: Selection of transcript expression in RNASeq from cortical MV extracted between P5 and P15 (Slaoui et al. 2023). [file CNS-31-e70614-s001.docx]

# Supplementary table 1

| Reagent or resource | Source | Reference |
| --- | --- | --- |
| **Antibodies** | | |
| P-gp (Western Blot) | Abcam | ab3366 |
| P-gp (IHC) | Sigma | 517312 |
| Claudin-5 | Thermo Fisher | 341600 |
| Histone 3 (Western Blot: 1/2000) | Ozyme | 14269S |
| anti-rabbit, HRP (WB : 1/2500) | Cohesion | CSA2115 |
| anti-mouse, HRP (WB : 1/2500) | Cohesion | CSA2108 |
| **qPCR Primers** | | |
| *Cldn5*  Forward TAAGGCACGGGTAGCACTCA  Reverse GGACAACGATGTTGGCGAAC | Sigma |  |
| *Abcb1a*  Forward GATAGGCTGGTTTGATGTGC  Reverse TCACAAGGGTTAGCTTCCAG | Sigma |  |
| *Slco1a4*  Forward GCCAGTGCAAGGCCAGAACC  Reverse AGGGTGGGGGTTTGCTACAGAA | Sigma |  |
| *Gapdh*  Forward AGGTCGGTGTGAACGGATTTG  Reverse TGTAGACCATGTAGTTGAGGTCA | Sigma |  |
| **Software and Algorithms** | | |
| ImageJ | https://imagej.nih.gov/ij/ |  |
| QuPath | https://qupath.github.io/ |  |

# Supplementary table 2

|  | **Fig. 1** | | | | | | | |
| --- | --- | --- | --- | --- | --- | --- | --- | --- |
|  | | | | | | Kruskal-Wallis test | Kruskal-Wallis test | Mann Whitney test (one-tailed) |
|  | Number of values | Median | Mean | Std. Deviation | Std. Error of Mean |  |  | 15-39 wg |
| 15-39 wg | 6 | 0.30 | 0.30 | 0.034 | 0.014 | 0.0042 |  |  |
| 0-2 years | 5 | 0.42 | 0.42 | 0.027 | 0.012 |  | 0.11 | 0.0043 |
| 2-5 years | 4 | 0.36 | 0.37 | 0.036 | 0.018 |  |  | 0.024 |
| >10 years | 5 | 0.43 | 0.42 | 0.036 | 0.016 |  |  | 0.0043 |

|  | **Fig. 2A** | | | | | | | | | | | |
| --- | --- | --- | --- | --- | --- | --- | --- | --- | --- | --- | --- | --- |
|  |  |  | | | | | | Kruskal-Wallis test | Mann Whitney test (one-tailed) | |  | |
| qPCR (normalisation with *Gapdh*) | Slco1a4 |  | Number of values | Median | Mean | Std. Deviation | Std. Error of Mean |  | P5 | P10 |  |  |
|  |  | P5 | 5 | 1.70 | 1.62 | 0.53 | 0.24 | <0.0001 |  |  |  |  |
|  |  | P10 | 4 | 4.94 | 4.90 | 0.78 | 0.39 |  | 0.0079 |  |  |  |
|  |  | P15 | 4 | 17.88 | 18.46 | 7.22 | 3.61 |  | 0.0079 | 0.014 |  |  |
|  |  | P5 - normalized | 5 | 1.05 | 1.00 | 0.33 | 0.15 |  |  |  |  |  |
|  |  | P10 - normalized | 4 | 3.05 | 3.02 | 0.48 | 0.24 |  |  |  |  |  |
|  |  | P15 - normalized | 4 | 11.04 | 11.40 | 4.46 | 2.23 |  |  |  |  |  |
|  |  | | | | | | | | | |  | |
|  | Abcb1a |  | Number of values | Median | Mean | Std. Deviation | Std. Error of Mean |  | P5 | P10 |  |  |
|  |  | P5 | 5 | 0.86 | 0.95 | 0.80 | 0.36 | <0.0001 |  |  |  |  |
|  |  | P10 | 4 | 7.39 | 7.19 | 0.80 | 0.40 |  | 0.0079 |  |  |  |
|  |  | P15 | 4 | 13.54 | 13.37 | 2.32 | 1.16 |  | 0.0079 | 0.014 |  |  |
|  |  | P5 - normalized | 5 | 0.90 | 1.00 | 0.85 | 0.38 |  |  |  |  |  |
|  |  | P10 - normalized | 4 | 7.76 | 7.55 | 0.84 | 0.42 |  |  |  |  |  |
|  |  | P15 - normalized | 4 | 14.22 | 14.04 | 2.43 | 1.22 |  |  |  |  |  |
|  |  |  |  |  |  |  |  |  |  |  |  |  |
|  | Cldn5 |  | Number of values | Median | Mean | Std. Deviation | Std. Error of Mean |  | P5 | P10 |  |  |
|  |  | P5 | 5 | 7.01 | 7.97 | 5.67 | 2.54 | 0.0025 |  |  |  |  |
|  |  | P10 | 4 | 41.14 | 38.15 | 9.43 | 4.71 |  | 0.0079 |  |  |  |
|  |  | P15 | 4 | 40.30 | 49.74 | 26.45 | 13.22 |  | 0.0079 | 0.3429 |  |  |
|  |  | P5 - normalized | 5 | 0.88 | 1.00 | 0.71 | 0.32 |  |  |  |  |  |
|  |  | P10 - normalized | 4 | 5.17 | 4.79 | 1.18 | 0.59 |  |  |  |  |  |
|  |  | P15 - normalized | 4 | 5.06 | 6.24 | 3.32 | 1.66 |  |  |  |  |  |
|  |  |  |  |  |  |  |  |  |  |  |  |  |
| qPCR (normalisation with *Ocln*) | Slco1a4 |  | Number of values | Median | Mean | Std. Deviation | Std. Error of Mean |  | P5 | P10 |  |  |
|  |  | P5 | 5 | 0.95 | 1.12 | 0.35 | 0.16 | 0.011 |  |  |  |  |
|  |  | P10 | 4 | 1.06 | 1.033 | 0.17 | 0.083 |  | 0.5 |  |  |  |
|  |  | P15 | 4 | 2.50 | 2.51 | 0.81 | 0.40 |  | 0.0079 | 0.014 |  |  |
|  |  | P5 - normalized | 5 | 0.85 | 1.00 | 0.31 | 0.14 |  |  |  |  |  |
|  |  | P10 - normalized | 4 | 0.95 | 0.93 | 0.15 | 0.074 |  |  |  |  |  |
|  |  | P15 - normalized | 4 | 2.24 | 2.25 | 0.72 | 0.36 |  |  |  |  |  |
|  |  | | | | | | | | | |  | |
|  | Abcb1a |  | Number of values | Median | Mean | Std. Deviation | Std. Error of Mean |  | P5 | P10 |  |  |
|  |  | P5 | 5 | 0.0048 | 0.0062 | 0.0054 | 0.0024 | 0.0032 |  |  |  |  |
|  |  | P10 | 4 | 0.053 | 0.068 | 0.045 | 0.023 |  | 0.0079 |  |  |  |
|  |  | P15 | 4 | 0.073 | 0.073 | 0.050 | 0.025 |  | 0.0079 | 0.500 |  |  |
|  |  | P5 - normalized | 5 | 0.78 | 1.00 | 0.87 | 0.39 |  |  |  |  |  |
|  |  | P10 - normalized | 4 | 8.54 | 11.00 | 7.30 | 3.65 |  |  |  |  |  |
|  |  | P15 - normalized | 4 | 11.82 | 11.91 | 8.10 | 4.05 |  |  |  |  |  |
|  |  |  |  |  |  |  |  |  |  |  |  |  |
|  | Cldn5 |  | Number of values | Median | Mean | Std. Deviation | Std. Error of Mean |  | P5 | P10 |  |  |
|  |  | P5 | 5 | 0.027 | 0.049 | 0.033 | 0.015 | 0.018 |  |  |  |  |
|  |  | P10 | 4 | 0.30 | 0.33 | 0.16 | 0.080 |  | 0.0079 |  |  |  |
|  |  | P15 | 4 | 0.26 | 0.31 | 0.27 | 0.14 |  | 0.032 | 0.44 |  |  |
|  |  | P5 - normalized | 5 | 0.56 | 1.00 | 0.68 | 0.30 |  |  |  |  |  |
|  |  | P10 - normalized | 4 | 6.19 | 6.76 | 3.26 | 1.63 |  |  |  |  |  |
|  |  | P15 - normalized | 4 | 5.28 | 6.23 | 5.57 | 2.79 |  |  |  |  |  |
|  |  |  |  |  |  |  |  |  |  |  |  |  |
|  | **Fig. 2B** | | | | | | | | | | | |
| WB | Claudin-5 |  | | | | | | Kruskal-Wallis test | Mann Whitney test (one-tailed) | | | |
|  |  |  | Number of values | Median | Mean | Std. Deviation | Std. Error of Mean |  | P5 | P15 | P30 |  |
|  |  | P5 | 4 | 1.00 | 1.00 | 0.26 | 0.13 | <0.0001 |  |  |  |  |
|  |  | P15 | 4 | 1.55 | 1.55 | 0.35 | 0.18 |  | 0.043 |  |  |  |
|  |  | P30 | 4 | 2.95 | 2.90 | 0.57 | 0.29 |  | 0.014 | 0.014 |  |  |
|  |  | P60 | 3 | 6.00 | 6.13 | 0.81 | 0.47 |  | 0.029 | 0.029 | 0.029 |  |
|  | P-gP |  | | | | | | Kruskal-Wallis test | Mann Whitney test (one-tailed) | | | |
|  |  |  | Number of values | Median | Mean | Std. Deviation | Std. Error of Mean |  | P5 | P15 | P30 |  |
|  |  | P5 | 4 | 0.00 | 0.00 | 0.00 | 0.00 | <0.0001 |  |  |  |  |
|  |  | P15 | 4 | 0.95 | 1.00 | 0.14 | 0.071 |  | 0.014 |  |  |  |
|  |  | P30 | 4 | 11.40 | 10.38 | 2.26 | 1.13 |  | 0.014 | 0.014 |  |  |
|  |  | P60 | 4 | 17.00 | 18.05 | 4.93 | 2.47 |  | 0.014 | 0.014 | 0.014 |  |

| **Fig. 3 and 4** | | | | | | | | | | |
| --- | --- | --- | --- | --- | --- | --- | --- | --- | --- | --- |
|  | | | | | | | | Kruskal-Wallis test | Mann Whitney test (one-tailed) | |
|  | | | Number of values | Median | Mean | Std. Deviation | Std. Error of Mean |  | P5 | P15 |
| 14C-Sucrose | Vv (µL/g) | P5 | 8 | 23.48 | 27.65 | 10.60 | 3.75 | 0.66 |  |  |
|  |  | P15 | 7 | 25.58 | 27.19 | 7.58 | 2.87 |  | 0.39 |  |
|  |  | P30 | 6 | 29.20 | 27.93 | 8.69 | 3.55 |  | 0.29 | 0.18 |
|  |  |  |  |  |  |  |  |  |  |  |
| 3H-Vérapamil | Kin (µL min-1 g-1) | P15 | 7 | 108.70 | 110.80 | 22.80 | 8.62 |  |  |  |
|  |  | P30 | 6 | 79.09 | 75.71 | 9.27 | 3.78 |  |  | 0.011 |
|  |  |  |  |  |  |  |  |  |  |  |
| 3H-Rosuvastatin | Kin (µL min-1 g-1) | P15 | 7 | 11.66 | 11.38 | 4.81 | 1.82 |  |  |  |
|  |  | P30 | 6 | 1.47 | 1.66 | 1.46 | 0.60 |  |  | 0.00060 |

# Supplementary table 3

| **Ensembl reference name** | **id** | **Gene symbol** | **Description** | **baseMean P5** | **baseMean P15** | **log2FoldChange (P15 v. P5)** | **padj** |
| --- | --- | --- | --- | --- | --- | --- | --- |
| ENSMUSG00000041378 | GSMG0014759 | Cldn5 | claudin 5 | 47649.68 | 52115.28 | 0.13 | 6.49E-01 |
| ENSMUSG00000020717 | GSMG0007522 | Pecam1 | platelet/endothelial cell adhesion molecule 1 | 9948.59 | 10823.02 | 0.12 | 5.62E-01 |
| ENSMUSG00000031871 | GSMG0037685 | Cdh5 | cadherin 5 | 2504.42 | 2589.02 | 0.05 | 9.03E-01 |
| ENSMUSG00000021638 | GSMG0011033 | Ocln | occludin | 4120.37 | 7244.67 | 0.81 | 4.65E-08 |
| ENSMUSG00000030516 | GSMG0036068 | Tjp1 | tight junction protein 1 | 2711.58 | 2596.58 | -0.06 | 8.18E-01 |
| ENSMUSG00000053062 | GSMG0015251 | Jam2 | junction adhesion molecule 2 | 1852.30 | 2081.97 | 0.17 | 4.25E-01 |

| **Ensembl reference name** | **id** | **Gene symbol** | **Description** | **baseMean P5** | **baseMean P15** | **log2FoldChange (P15 v. P5)** | **padj** | **Specific to Endothelial cells (Slaoui, Gilbert et al. 2023)** |
| --- | --- | --- | --- | --- | --- | --- | --- | --- |
| ENSMUSG00000015243 | GSMG0027811 | Abca1 | ATP-binding cassette, sub-family A (ABC1), member 1 | 9933.83 | 5789.72 | -0.78 | 1.79E-03 |  |
| ENSMUSG00000026944 | GSMG0020799 | Abca2 | ATP-binding cassette, sub-family A (ABC1), member 2 | 843.21 | 1167.89 | 0.47 | 5.56E-02 |  |
| ENSMUSG00000024130 | GSMG0016337 | Abca3 | ATP-binding cassette, sub-family A (ABC1), member 3 | 488.94 | 390.77 | -0.32 | 2.41E-01 |  |
| ENSMUSG00000018800 | GSMG0007579 | Abca5 | ATP-binding cassette, sub-family A (ABC1), member 5 | 558.47 | 434.91 | -0.36 | 1.40E-01 |  |
| ENSMUSG00000035722 | GSMG0003244 | Abca7 | ATP-binding cassette, sub-family A (ABC1), member 7 | 337.84 | 516.91 | 0.61 | 8.11E-03 |  |
| ENSMUSG00000041797 | GSMG0007573 | Abca9 | ATP-binding cassette, sub-family A (ABC1), member 9 | 462.94 | 743.90 | 0.68 | 1.02E-04 |  |
| ENSMUSG00000031974 | GSMG0039053 | Abcb10 | ATP-binding cassette, sub-family B (MDR/TAP), member 10 | 1280.80 | 971.27 | -0.40 | 4.03E-02 |  |
| ENSMUSG00000040584 | GSMG0028802 | Abcb1a | ATP-binding cassette, sub-family B (MDR/TAP), member 1A | 1612.23 | 3746.35 | 1.22 | 7.19E-15 | YES |
| ENSMUSG00000026198 | GSMG0001768 | Abcb6 | ATP-binding cassette, sub-family B (MDR/TAP), member 6 | 442.17 | 348.75 | -0.34 | 1.98E-01 |  |
| ENSMUSG00000031333 | GSMG0042764 | Abcb7 | ATP-binding cassette, sub-family B (MDR/TAP), member 7 | 235.36 | 194.64 | -0.27 | 3.92E-01 |  |
| ENSMUSG00000028973 | GSMG0028918 | Abcb8 | ATP-binding cassette, sub-family B (MDR/TAP), member 8 | 2402.64 | 1274.25 | -0.91 | 1.17E-06 |  |
| ENSMUSG00000029408 | GSMG0031078 | Abcb9 | ATP-binding cassette, sub-family B (MDR/TAP), member 9 | 554.75 | 736.48 | 0.41 | 3.95E-02 |  |
| ENSMUSG00000023088 | GSMG0014706 | Abcc1 | ATP-binding cassette, sub-family C (CFTR/MRP), member 1 | 340.44 | 356.18 | 0.07 | 8.72E-01 |  |
| ENSMUSG00000032842 | GSMG0017594 | Abcc10 | ATP-binding cassette, sub-family C (CFTR/MRP), member 10 | 312.40 | 375.69 | 0.27 | 3.89E-01 |  |
| ENSMUSG00000032849 | GSMG0012855 | Abcc4 | ATP-binding cassette, sub-family C (CFTR/MRP), member 4 | 3096.86 | 5596.56 | 0.85 | 7.66E-10 |  |
| ENSMUSG00000022822 | GSMG0015543 | Abcc5 | ATP-binding cassette, sub-family C (CFTR/MRP), member 5 | 370.75 | 431.97 | 0.22 | 4.64E-01 |  |
| ENSMUSG00000030834 | GSMG0035929 | Abcc6 | ATP-binding cassette, sub-family C (CFTR/MRP), member 6 | 2061.14 | 3817.48 | 0.89 | 2.98E-06 |  |
| ENSMUSG00000040136 | GSMG0035931 | Abcc8 | ATP-binding cassette, sub-family C (CFTR/MRP), member 8 | 248.91 | 387.23 | 0.64 | 1.21E-02 |  |
| ENSMUSG00000030249 | GSMG0033855 | Abcc9 | ATP-binding cassette, sub-family C (CFTR/MRP), member 9 | 3439.74 | 2204.11 | -0.64 | 1.09E-03 |  |
| ENSMUSG00000031378 | GSMG0041843 | Abcd1 | ATP-binding cassette, sub-family D (ALD), member 1 | 254.37 | 389.77 | 0.62 | 9.23E-03 |  |
| ENSMUSG00000055782 | GSMG0014395 | Abcd2 | ATP-binding cassette, sub-family D (ALD), member 2 | 105.33 | 179.84 | 0.78 | 5.91E-03 |  |
| ENSMUSG00000028127 | GSMG0025996 | Abcd3 | ATP-binding cassette, sub-family D (ALD), member 3 | 1503.44 | 1360.75 | -0.14 | 5.28E-01 |  |
| ENSMUSG00000021240 | GSMG0009118 | Abcd4 | ATP-binding cassette, sub-family D (ALD), member 4 | 423.12 | 527.20 | 0.32 | 2.02E-01 |  |
| ENSMUSG00000058355 | GSMG0038639 | Abce1 | ATP-binding cassette, sub-family E (OABP), member 1 | 3033.39 | 2325.38 | -0.38 | 6.36E-02 |  |
| ENSMUSG00000038762 | GSMG0017482 | Abcf1 | ATP-binding cassette, sub-family F (GCN20), member 1 | 3859.26 | 3161.88 | -0.29 | 8.77E-02 |  |
| ENSMUSG00000028953 | GSMG0030261 | Abcf2 | ATP-binding cassette, sub-family F (GCN20), member 2 | 2447.10 | 2292.93 | -0.09 | 7.14E-01 |  |
| ENSMUSG00000003234 | GSMG0014775 | Abcf3 | ATP-binding cassette, sub-family F (GCN20), member 3 | 2314.48 | 2528.88 | 0.13 | 5.42E-01 |  |
| ENSMUSG00000024030 | GSMG0016480 | Abcg1 | ATP-binding cassette, sub-family G (WHITE), member 1 | 767.77 | 744.28 | -0.04 | 9.04E-01 |  |
| ENSMUSG00000029802 | GSMG0031959 | Abcg2 | ATP-binding cassette, sub-family G (WHITE), member 2 | 7014.99 | 9315.40 | 0.41 | 2.99E-02 | YES |
| ENSMUSG00000032131 | GSMG0040650 | Abcg4 | ATP-binding cassette, sub-family G (WHITE), member 4 | 594.54 | 423.19 | -0.49 | 4.37E-02 |  |

| **Ensembl reference name** | **id** | **Gene symbol** | **Description** | **baseMean P5** | **baseMean P15** | **log2FoldChange (P15 v. P5)** | **padj** | **Specific to Endothelial cells (Slaoui, Gilbert et al. 2023)** |
| --- | --- | --- | --- | --- | --- | --- | --- | --- |
| ENSMUSG00000024327,ENSMUSG00000073422 | GSMG0017420 | H2-Ke6 // Slc39a7 | H2-K region expressed gene 6 // solute carrier family 39 (zinc transporter), member 7 | 2728.03 | 2471.78 | -0.14 | 5.44E-01 |  |
| ENSMUSG00000031684 | GSMG0037484 | Slc10a7 | solute carrier family 10 (sodium/bile acid cotransporter family), member 7 | 251.83 | 257.14 | 0.03 | 9.44E-01 |  |
| ENSMUSG00000023030 | GSMG0014508 | Slc11a2 | solute carrier family 11 (proton-coupled divalent metal ion transporters), member 2 | 1437.02 | 1378.00 | -0.06 | 8.30E-01 |  |
| ENSMUSG00000024597 | GSMG0018432 | Slc12a2 | solute carrier family 12, member 2 | 1294.44 | 1337.79 | 0.05 | 8.90E-01 |  |
| ENSMUSG00000017765 | GSMG0038860 | Slc12a4 | solute carrier family 12, member 4 | 1729.33 | 2457.33 | 0.51 | 9.10E-03 |  |
| ENSMUSG00000017740 | GSMG0022165 | Slc12a5 | solute carrier family 12, member 5 | 808.06 | 899.33 | 0.15 | 6.84E-01 |  |
| ENSMUSG00000027130,ENSMUSG00000027132 | GSMG0021553 | Slc12a6 // Katnbl1 | solute carrier family 12, member 6 // katanin p80 subunit B like 1 | 4106.48 | 4506.67 | 0.13 | 5.39E-01 |  |
| ENSMUSG00000017756 | GSMG0009936 | Slc12a7 | solute carrier family 12, member 7 | 795.58 | 724.73 | -0.13 | 6.54E-01 |  |
| ENSMUSG00000037344 | GSMG0031234 | Slc12a9 | solute carrier family 12 (potassium/chloride transporters), member 9 | 581.50 | 591.25 | 0.02 | 9.48E-01 |  |
| ENSMUSG00000018459 | GSMG0023891 | Slc13a3 | solute carrier family 13 (sodium-dependent dicarboxylate transporter), member 3 | 597.92 | 444.56 | -0.43 | 8.07E-02 |  |
| ENSMUSG00000029843 | GSMG0032867 | Slc13a4 | solute carrier family 13 (sodium/sulfate symporters), member 4 | 676.42 | 671.19 | -0.01 | 9.86E-01 |  |
| ENSMUSG00000059336 | GSMG0019170 | Slc14a1 | solute carrier family 14 (urea transporter), member 1 | 92.19 | 83.12 | -0.15 | 7.79E-01 |  |
| ENSMUSG00000022899 | GSMG0015717 | Slc15a2 | solute carrier family 15 (H+/peptide transporter), member 2 | 146.57 | 92.33 | -0.65 | 7.68E-02 |  |
| ENSMUSG00000029416 | GSMG0031105 | Slc15a4 | solute carrier family 15, member 4 | 873.66 | 976.30 | 0.16 | 4.68E-01 |  |
| ENSMUSG00000032902 | GSMG0024772 | Slc16a1 | solute carrier family 16 (monocarboxylic acid transporters), member 1 | 79908.91 | 135016.61 | 0.76 | 5.88E-06 | YES |
| ENSMUSG00000040938 | GSMG0005436 | Slc16a11 | solute carrier family 16 (monocarboxylic acid transporters), member 11 | 552.16 | 721.78 | 0.39 | 1.35E-01 |  |
| ENSMUSG00000009378 | GSMG0020325 | Slc16a12 | solute carrier family 16 (monocarboxylic acid transporters), member 12 | 545.22 | 569.10 | 0.06 | 8.46E-01 |  |
| ENSMUSG00000044367 | GSMG0006898 | Slc16a13 | solute carrier family 16 (monocarboxylic acid transporters), member 13 | 395.03 | 348.75 | -0.18 | 5.18E-01 |  |
| ENSMUSG00000033965 | GSMG0042756 | Slc16a2 | solute carrier family 16 (monocarboxylic acid transporters), member 2 | 1290.53 | 1793.87 | 0.48 | 1.63E-02 |  |
| ENSMUSG00000027896 | GSMG0024804 | Slc16a4 | solute carrier family 16 (monocarboxylic acid transporters), member 4 | 2508.89 | 3682.23 | 0.55 | 1.10E-02 | YES |
| ENSMUSG00000041920 | GSMG0007568 | Slc16a6 | solute carrier family 16 (monocarboxylic acid transporters), member 6 | 442.84 | 371.89 | -0.25 | 3.25E-01 |  |
| ENSMUSG00000020102 | GSMG0004717 | Slc16a7 | solute carrier family 16 (monocarboxylic acid transporters), member 7 | 169.74 | 126.64 | -0.42 | 1.67E-01 |  |
| ENSMUSG00000037762 | GSMG0003136 | Slc16a9 | solute carrier family 16 (monocarboxylic acid transporters), member 9 | 1155.36 | 1288.32 | 0.16 | 5.10E-01 |  |
| ENSMUSG00000049624 | GSMG0041053 | Slc17a5 | solute carrier family 17 (anion/sugar transporter), member 5 | 522.64 | 463.55 | -0.17 | 5.03E-01 |  |
| ENSMUSG00000030500 | GSMG0034535 | Slc17a6 | solute carrier family 17 (sodium-dependent inorganic phosphate cotransporter), member 6 | 300.02 | 68.95 | -2.11 | 5.50E-19 |  |
| ENSMUSG00000070570 | GSMG0034459 | Slc17a7 | solute carrier family 17 (sodium-dependent inorganic phosphate cotransporter), member 7 | 2639.02 | 7821.70 | 1.57 | 2.50E-17 |  |
| ENSMUSG00000025094 | GSMG0019887 | Slc18a2 | solute carrier family 18 (vesicular monoamine), member 2 | 467.11 | 400.72 | -0.23 | 5.14E-01 |  |
| ENSMUSG00000037455 | GSMG0002790 | Slc18b1 | solute carrier family 18, subfamily B, member 1 | 478.29 | 367.73 | -0.38 | 6.43E-02 |  |
| ENSMUSG00000001436 | GSMG0003189 | Slc19a1 | solute carrier family 19 (folate transporter), member 1 | 2958.05 | 3508.51 | 0.25 | 1.75E-01 |  |
| ENSMUSG00000040918 | GSMG0000983 | Slc19a2 | solute carrier family 19 (thiamine transporter), member 2 | 279.46 | 323.22 | 0.21 | 5.00E-01 |  |
| ENSMUSG00000038496 | GSMG0001823 | Slc19a3 | solute carrier family 19, member 3 | 477.69 | 1580.96 | 1.73 | 3.15E-26 |  |
| ENSMUSG00000024935 | GSMG0019565 | Slc1a1 | solute carrier family 1 (neuronal/epithelial high affinity glutamate transporter, system Xag), member 1 | 212.35 | 322.60 | 0.60 | 9.95E-03 |  |
| ENSMUSG00000005089 | GSMG0021475 | Slc1a2 | solute carrier family 1 (glial high affinity glutamate transporter), member 2 | 605.14 | 1873.20 | 1.63 | 2.40E-20 |  |
| ENSMUSG00000005360 | GSMG0013758 | Slc1a3 | solute carrier family 1 (glial high affinity glutamate transporter), member 3 | 5616.58 | 5146.15 | -0.13 | 5.44E-01 |  |
| ENSMUSG00000020142 | GSMG0006386 | Slc1a4 | solute carrier family 1 (glutamate/neutral amino acid transporter), member 4 | 5498.99 | 1081.83 | -2.34 | 2.30E-44 |  |
| ENSMUSG00000001918 | GSMG0034061 | Slc1a5 | solute carrier family 1 (neutral amino acid transporter), member 5 | 398.89 | 433.77 | 0.12 | 6.95E-01 |  |
| ENSMUSG00000027397 | GSMG0021770 | Slc20a1 | solute carrier family 20, member 1 | 462.47 | 363.45 | -0.34 | 1.21E-01 |  |
| ENSMUSG00000037656 | GSMG0037090 | Slc20a2 | solute carrier family 20, member 2 | 1923.31 | 1947.36 | 0.02 | 9.51E-01 |  |
| ENSMUSG00000022199 | GSMG0012406 | Slc22a17 | solute carrier family 22 (organic cation transporter), member 17 | 6703.44 | 6551.72 | -0.03 | 9.28E-01 |  |
| ENSMUSG00000038267 | GSMG0010530 | Slc22a23 | solute carrier family 22, member 23 | 306.93 | 150.61 | -1.02 | 2.18E-04 |  |
| ENSMUSG00000018900 | GSMG0006668 | Slc22a5 | solute carrier family 22 (organic cation transporter), member 5 | 414.65 | 489.13 | 0.24 | 3.99E-01 |  |
| ENSMUSG00000024650 | GSMG0019363 | Slc22a6 | solute carrier family 22 (organic anion transporter), member 6 | 566.08 | 566.26 | 0.00 | 9.95E-01 |  |
| ENSMUSG00000063796 | GSMG0019362 | Slc22a8 | solute carrier family 22 (organic anion transporter), member 8 | 20690.14 | 21843.02 | 0.08 | 7.24E-01 |  |
| ENSMUSG00000027340 | GSMG0023574 | Slc23a2 | solute carrier family 23 (nucleobase transporters), member 2 | 667.86 | 593.71 | -0.17 | 5.30E-01 |  |
| ENSMUSG00000037996 | GSMG0028023 | Slc24a2 | solute carrier family 24 (sodium/potassium/calcium exchanger), member 2 | 162.03 | 450.70 | 1.48 | 1.07E-11 |  |
| ENSMUSG00000063873 | GSMG0021904 | Slc24a3 | solute carrier family 24 (sodium/potassium/calcium exchanger), member 3 | 615.64 | 647.30 | 0.07 | 8.67E-01 |  |
| ENSMUSG00000041771 | GSMG0008451 | Slc24a4 | solute carrier family 24 (sodium/potassium/calcium exchanger), member 4 | 20.93 | 66.46 | 1.66 | 2.69E-03 |  |
| ENSMUSG00000035183 | GSMG0021713 | Slc24a5 | solute carrier family 24, member 5 | 151.13 | 101.95 | -0.57 | 5.82E-02 |  |
| ENSMUSG00000003528 | GSMG0015513 | Slc25a1 | solute carrier family 25 (mitochondrial carrier, citrate transporter), member 1 | 1391.29 | 1414.72 | 0.02 | 9.33E-01 |  |
| ENSMUSG00000025792 | GSMG0006215 | Slc25a10 | solute carrier family 25 (mitochondrial carrier, dicarboxylate transporter), member 10 | 489.17 | 361.82 | -0.43 | 4.89E-02 |  |
| ENSMUSG00000014606 | GSMG0006911 | Slc25a11 | solute carrier family 25 (mitochondrial carrier oxoglutarate carrier), member 11 | 1018.33 | 1051.03 | 0.05 | 8.70E-01 |  |
| ENSMUSG00000027010 | GSMG0022952 | Slc25a12 | solute carrier family 25 (mitochondrial carrier, Aralar), member 12 | 1574.07 | 1299.18 | -0.28 | 1.44E-01 |  |
| ENSMUSG00000031105 | GSMG0041733 | Slc25a14 | solute carrier family 25 (mitochondrial carrier, brain), member 14 | 325.03 | 306.96 | -0.08 | 8.01E-01 |  |
| ENSMUSG00000031482 | GSMG0038182 | Slc25a15 | solute carrier family 25 (mitochondrial carrier ornithine transporter), member 15 | 297.00 | 271.02 | -0.13 | 7.28E-01 |  |
| ENSMUSG00000071253 | GSMG0003074 | Slc25a16 | solute carrier family 25 (mitochondrial carrier, Graves disease autoantigen), member 16 | 274.91 | 283.84 | 0.05 | 9.13E-01 |  |
| ENSMUSG00000022404 | GSMG0014280 | Slc25a17 | solute carrier family 25 (mitochondrial carrier, peroxisomal membrane protein), member 17 | 911.60 | 757.97 | -0.27 | 2.20E-01 |  |
| ENSMUSG00000004902 | GSMG0032414 | Slc25a18 | solute carrier family 25 (mitochondrial carrier), member 18 | 313.86 | 222.28 | -0.49 | 3.37E-02 |  |
| ENSMUSG00000020744 | GSMG0043684 | Slc25a19 | solute carrier family 25 (mitochondrial thiamine pyrophosphate carrier), member 19 | 272.46 | 242.19 | -0.17 | 6.21E-01 |  |
| ENSMUSG00000032602 | GSMG0040100 | Slc25a20 | solute carrier family 25 (mitochondrial carnitine/acylcarnitine translocase), member 20 | 1174.05 | 962.95 | -0.28 | 1.77E-01 |  |
| ENSMUSG00000019082 | GSMG0036867 | Slc25a22 | solute carrier family 25 (mitochondrial carrier, glutamate), member 22 | 857.06 | 808.48 | -0.08 | 7.79E-01 |  |
| ENSMUSG00000046329 | GSMG0017715 | Slc25a23 | solute carrier family 25 (mitochondrial carrier%3B phosphate carrier), member 23 | 2100.57 | 2508.71 | 0.26 | 3.18E-01 |  |
| ENSMUSG00000040322 | GSMG0024830 | Slc25a24 | solute carrier family 25 (mitochondrial carrier, phosphate carrier), member 24 | 260.16 | 297.72 | 0.20 | 4.97E-01 |  |
| ENSMUSG00000026819 | GSMG0022642 | Slc25a25 | solute carrier family 25 (mitochondrial carrier, phosphate carrier), member 25 | 429.98 | 583.06 | 0.44 | 7.02E-02 |  |
| ENSMUSG00000045100 | GSMG0032243 | Slc25a26 | solute carrier family 25 (mitochondrial carrier, phosphate carrier), member 26 | 100.20 | 101.75 | 0.03 | 9.63E-01 |  |
| ENSMUSG00000023912 | GSMG0017560 | Slc25a27 | solute carrier family 25, member 27 | 178.24 | 165.73 | -0.11 | 7.99E-01 |  |
| ENSMUSG00000040414 | GSMG0020416 | Slc25a28 | solute carrier family 25, member 28 | 1046.18 | 1210.91 | 0.21 | 4.98E-01 |  |
| ENSMUSG00000021265 | GSMG0009305 | Slc25a29 | solute carrier family 25 (mitochondrial carrier, palmitoylcarnitine transporter), member 29 | 381.70 | 320.03 | -0.25 | 3.90E-01 |  |
| ENSMUSG00000061904 | GSMG0004454 | Slc25a3 | solute carrier family 25 (mitochondrial carrier, phosphate carrier), member 3 | 4902.02 | 5306.66 | 0.11 | 6.07E-01 |  |
| ENSMUSG00000022003 | GSMG0012671 | Slc25a30 | solute carrier family 25, member 30 | 119.27 | 106.91 | -0.15 | 7.63E-01 |  |
| ENSMUSG00000022299 | GSMG0013904 | Slc25a32 | solute carrier family 25, member 32 | 170.90 | 270.48 | 0.66 | 9.44E-03 |  |
| ENSMUSG00000028982 | GSMG0028678 | Slc25a33 | solute carrier family 25, member 33 | 237.30 | 393.12 | 0.73 | 3.51E-04 |  |
| ENSMUSG00000018740 | GSMG0005386 | Slc25a35 | solute carrier family 25, member 35 | 90.04 | 175.38 | 0.96 | 1.25E-03 |  |
| ENSMUSG00000032449 | GSMG0041167 | Slc25a36 | solute carrier family 25, member 36 | 1911.12 | 1664.32 | -0.20 | 3.73E-01 |  |
| ENSMUSG00000034248 | GSMG0012592 | Slc25a37 | solute carrier family 25, member 37 | 1054.71 | 1155.49 | 0.13 | 6.00E-01 |  |
| ENSMUSG00000032519 | GSMG0040233 | Slc25a38 | solute carrier family 25, member 38 | 331.40 | 347.96 | 0.07 | 8.32E-01 |  |
| ENSMUSG00000018677 | GSMG0007447 | Slc25a39 | solute carrier family 25, member 39 | 3194.19 | 2903.36 | -0.14 | 5.04E-01 |  |
| ENSMUSG00000031633 | GSMG0038369 | Slc25a4 | solute carrier family 25 (mitochondrial carrier, adenine nucleotide translocator), member 4 | 5111.00 | 5064.35 | -0.01 | 9.63E-01 |  |
| ENSMUSG00000054099 | GSMG0028800 | Slc25a40 | solute carrier family 25, member 40 | 188.26 | 200.68 | 0.09 | 8.53E-01 |  |
| ENSMUSG00000002346 | GSMG0038544 | Slc25a42 | solute carrier family 25, member 42 | 526.27 | 512.75 | -0.04 | 9.16E-01 |  |
| ENSMUSG00000050144 | GSMG0025624 | Slc25a44 | solute carrier family 25, member 44 | 432.51 | 410.70 | -0.07 | 8.47E-01 |  |
| ENSMUSG00000024818 | GSMG0019320 | Slc25a45 | solute carrier family 25, member 45 | 182.80 | 215.48 | 0.24 | 5.13E-01 |  |
| ENSMUSG00000024259 | GSMG0018800 | Slc25a46 | solute carrier family 25, member 46 | 1978.40 | 1939.88 | -0.03 | 9.19E-01 |  |
| ENSMUSG00000016319 | GSMG0041668 | Slc25a5 | solute carrier family 25 (mitochondrial carrier, adenine nucleotide translocator), member 5 | 5119.72 | 4143.44 | -0.30 | 9.07E-02 |  |
| ENSMUSG00000045973 | GSMG0027763 | Slc25a51 | solute carrier family 25, member 51 | 1659.42 | 1318.47 | -0.33 | 1.85E-01 |  |
| ENSMUSG00000044348 | GSMG0042867 | Slc25a53 | solute carrier family 25, member 53 | 114.94 | 109.87 | -0.07 | 8.90E-01 |  |
| ENSMUSG00000040441 | GSMG0043694 | Slc26a10 | solute carrier family 26, member 10 | 41.54 | 357.59 | 3.11 | 2.25E-34 |  |
| ENSMUSG00000039908 | GSMG0006184 | Slc26a11 | solute carrier family 26, member 11 | 268.67 | 291.15 | 0.12 | 7.49E-01 |  |
| ENSMUSG00000034320 | GSMG0019017 | Slc26a2 | solute carrier family 26 (sulfate transporter), member 2 | 272.70 | 233.64 | -0.23 | 4.72E-01 |  |
| ENSMUSG00000023259 | GSMG0040107 | Slc26a6 | solute carrier family 26, member 6 | 373.99 | 491.24 | 0.40 | 1.22E-01 |  |
| ENSMUSG00000031808 | GSMG0037425 | Slc27a1 | solute carrier family 27 (fatty acid transporter), member 1 | 1452.39 | 1529.60 | 0.07 | 8.30E-01 |  |
| ENSMUSG00000027932 | GSMG0025665 | Slc27a3 | solute carrier family 27 (fatty acid transporter), member 3 | 284.39 | 121.04 | -1.24 | 3.48E-08 |  |
| ENSMUSG00000059316 | GSMG0020874 | Slc27a4 | solute carrier family 27 (fatty acid transporter), member 4 | 656.28 | 622.21 | -0.08 | 8.44E-01 |  |
| ENSMUSG00000023942 | GSMG0017577 | Slc29a1 | solute carrier family 29 (nucleoside transporters), member 1 | 5952.24 | 2994.72 | -0.99 | 4.29E-13 |  |
| ENSMUSG00000024891 | GSMG0019288 | Slc29a2 | solute carrier family 29 (nucleoside transporters), member 2 | 381.88 | 195.10 | -0.97 | 9.21E-05 |  |
| ENSMUSG00000020100 | GSMG0004136 | Slc29a3 | solute carrier family 29 (nucleoside transporters), member 3 | 72.30 | 98.18 | 0.44 | 3.29E-01 |  |
| ENSMUSG00000050822 | GSMG0051995 | Slc29a4 | solute carrier family 29 (nucleoside transporters), member 4 | 2940.16 | 1775.65 | -0.73 | 1.07E-05 |  |
| ENSMUSG00000028645 | GSMG0026997 | Slc2a1 | solute carrier family 2 (facilitated glucose transporter), member 1 | 14860.71 | 31397.21 | 1.08 | 5.15E-11 |  |
| ENSMUSG00000037490 | GSMG0002785 | Slc2a12 | solute carrier family 2 (facilitated glucose transporter), member 12 | 212.91 | 149.66 | -0.50 | 1.35E-01 |  |
| ENSMUSG00000036298 | GSMG0014396 | Slc2a13 | solute carrier family 2 (facilitated glucose transporter), member 13 | 206.85 | 281.61 | 0.45 | 1.35E-01 |  |
| ENSMUSG00000003153 | GSMG0033645 | Slc2a3 | solute carrier family 2 (facilitated glucose transporter), member 3 | 1021.98 | 1570.15 | 0.62 | 4.36E-04 |  |
| ENSMUSG00000018566 | GSMG0006894 | Slc2a4 | solute carrier family 2 (facilitated glucose transporter), member 4 | 84.01 | 152.93 | 0.86 | 4.62E-03 |  |
| ENSMUSG00000036067 | GSMG0022576 | Slc2a6 | solute carrier family 2 (facilitated glucose transporter), member 6 | 399.14 | 527.81 | 0.40 | 1.19E-01 |  |
| ENSMUSG00000026791 | GSMG0022653 | Slc2a8 | solute carrier family 2, (facilitated glucose transporter), member 8 | 1146.64 | 1508.78 | 0.40 | 4.12E-02 |  |
| ENSMUSG00000037434 | GSMG0001242 | Slc30a1 | solute carrier family 30 (zinc transporter), member 1 | 2549.41 | 3162.21 | 0.31 | 2.84E-01 |  |
| ENSMUSG00000026614 | GSMG0001197 | Slc30a10 | solute carrier family 30, member 10 | 2276.69 | 2116.09 | -0.11 | 6.55E-01 |  |
| ENSMUSG00000029151 | GSMG0030319 | Slc30a3 | solute carrier family 30 (zinc transporter), member 3 | 189.37 | 1030.15 | 2.44 | 2.25E-30 |  |
| ENSMUSG00000005802 | GSMG0023456 | Slc30a4 | solute carrier family 30 (zinc transporter), member 4 | 226.79 | 255.64 | 0.17 | 6.40E-01 |  |
| ENSMUSG00000021629 | GSMG0011042 | Slc30a5 | solute carrier family 30 (zinc transporter), member 5 | 1356.63 | 1380.27 | 0.03 | 9.34E-01 |  |
| ENSMUSG00000024069 | GSMG0016921 | Slc30a6 | solute carrier family 30 (zinc transporter), member 6 | 407.13 | 443.94 | 0.12 | 6.84E-01 |  |
| ENSMUSG00000054414 | GSMG0025966 | Slc30a7 | solute carrier family 30 (zinc transporter), member 7 | 486.12 | 452.97 | -0.10 | 7.23E-01 |  |
| ENSMUSG00000029221 | GSMG0029247 | Slc30a9 | solute carrier family 30 (zinc transporter), member 9 | 914.93 | 942.30 | 0.04 | 8.91E-01 |  |
| ENSMUSG00000066150 | GSMG0026620 | Slc31a1 | solute carrier family 31, member 1 | 3014.40 | 3702.21 | 0.30 | 1.05E-01 |  |
| ENSMUSG00000066152 | GSMG0026619 | Slc31a2 | solute carrier family 31, member 2 | 219.47 | 236.02 | 0.10 | 7.83E-01 |  |
| ENSMUSG00000027822 | GSMG0025472 | Slc33a1 | solute carrier family 33 (acetyl-CoA transporter), member 1 | 271.89 | 260.36 | -0.06 | 8.88E-01 |  |
| ENSMUSG00000028293 | GSMG0027651 | Slc35a1 | solute carrier family 35 (CMP-sialic acid transporter), member 1 | 642.59 | 721.19 | 0.17 | 4.92E-01 |  |
| ENSMUSG00000031156 | GSMG0041568 | Slc35a2 | solute carrier family 35 (UDP-galactose transporter), member A2 | 734.60 | 793.13 | 0.11 | 6.81E-01 |  |
| ENSMUSG00000027957 | GSMG0025974 | Slc35a3 | solute carrier family 35 (UDP-N-acetylglucosamine (UDP-GlcNAc) transporter), member 3 | 387.32 | 386.80 | 0.00 | 9.98E-01 |  |
| ENSMUSG00000033272 | GSMG0018258 | Slc35a4 | solute carrier family 35, member A4 | 3530.51 | 3226.83 | -0.13 | 5.88E-01 |  |
| ENSMUSG00000022664 | GSMG0015760 | Slc35a5 | solute carrier family 35, member A5 | 381.76 | 321.18 | -0.25 | 3.46E-01 |  |
| ENSMUSG00000020873 | GSMG0005803 | Slc35b1 | solute carrier family 35, member B1 | 2659.46 | 2307.32 | -0.20 | 2.82E-01 |  |
| ENSMUSG00000037089 | GSMG0016711 | Slc35b2 | solute carrier family 35, member B2 | 2790.38 | 2661.45 | -0.07 | 8.03E-01 |  |
| ENSMUSG00000021432 | GSMG0010567 | Slc35b3 | solute carrier family 35, member B3 | 917.20 | 814.47 | -0.17 | 4.63E-01 |  |
| ENSMUSG00000018999 | GSMG0032847 | Slc35b4 | solute carrier family 35, member B4 | 373.63 | 371.77 | 0.00 | 9.93E-01 |  |
| ENSMUSG00000049922 | GSMG0023195 | Slc35c1 | solute carrier family 35, member C1 | 237.93 | 301.36 | 0.34 | 2.92E-01 |  |
| ENSMUSG00000017664 | GSMG0023886 | Slc35c2 | solute carrier family 35, member C2 | 717.23 | 626.58 | -0.19 | 4.05E-01 |  |
| ENSMUSG00000028521 | GSMG0028131 | Slc35d1 | solute carrier family 35 (UDP-glucuronic acid/UDP-N-acetylgalactosamine dual transporter), member D1 | 145.94 | 200.91 | 0.46 | 9.73E-02 |  |
| ENSMUSG00000033114 | GSMG0010798 | Slc35d2 | solute carrier family 35, member D2 | 120.56 | 159.85 | 0.40 | 1.96E-01 |  |
| ENSMUSG00000019731 | GSMG0038602 | Slc35e1 | solute carrier family 35, member E1 | 301.24 | 299.75 | -0.01 | 9.87E-01 |  |
| ENSMUSG00000042202 | GSMG0027486 | Slc35e2 | solute carrier family 35, member E2 | 168.47 | 141.66 | -0.25 | 5.22E-01 |  |
| ENSMUSG00000060181 | GSMG0004643 | Slc35e3 | solute carrier family 35, member E3 | 805.68 | 634.93 | -0.34 | 6.77E-02 |  |
| ENSMUSG00000048807 | GSMG0006264 | Slc35e4 | solute carrier family 35, member E4 | 804.94 | 1165.34 | 0.53 | 1.18E-02 |  |
| ENSMUSG00000038602 | GSMG0002988 | Slc35f1 | solute carrier family 35, member F1 | 733.60 | 277.59 | -1.40 | 1.32E-13 |  |
| ENSMUSG00000042195 | GSMG0039538 | Slc35f2 | solute carrier family 35, member F2 | 1819.09 | 1653.01 | -0.14 | 5.12E-01 |  |
| ENSMUSG00000057060 | GSMG0038002 | Slc35f3 | solute carrier family 35, member F3 | 147.67 | 148.37 | 0.01 | 9.91E-01 |  |
| ENSMUSG00000021852 | GSMG0012335 | Slc35f4 | solute carrier family 35, member F4 | 172.43 | 110.75 | -0.64 | 2.45E-02 |  |
| ENSMUSG00000026342 | GSMG0000705 | Slc35f5 | solute carrier family 35, member F5 | 1236.51 | 1281.44 | 0.05 | 8.63E-01 |  |
| ENSMUSG00000029175 | GSMG0028993 | Slc35f6 | solute carrier family 35, member F6 | 277.67 | 368.46 | 0.41 | 1.44E-01 |  |
| ENSMUSG00000070287 | GSMG0052521 | Slc35g2 | solute carrier family 35, member G2 | 113.55 | 182.78 | 0.69 | 1.53E-02 |  |
| ENSMUSG00000043885 | GSMG0039170 | Slc36a4 | solute carrier family 36 (proton/amino acid symporter), member 4 | 432.77 | 438.57 | 0.02 | 9.55E-01 |  |
| ENSMUSG00000024036 | GSMG0016482 | Slc37a1 | solute carrier family 37 (glycerol-3-phosphate transporter), member 1 | 186.28 | 295.16 | 0.66 | 1.38E-02 |  |
| ENSMUSG00000029924 | GSMG0032920 | Slc37a3 | solute carrier family 37 (glycerol-3-phosphate transporter), member 3 | 828.90 | 757.88 | -0.13 | 6.14E-01 |  |
| ENSMUSG00000032114 | GSMG0039436 | Slc37a4 | solute carrier family 37 (glucose-6-phosphate transporter), member 4 | 607.35 | 599.90 | -0.02 | 9.62E-01 |  |
| ENSMUSG00000023169 | GSMG0014444 | Slc38a1 | solute carrier family 38, member 1 | 1079.68 | 616.44 | -0.81 | 1.77E-05 |  |
| ENSMUSG00000061306 | GSMG0007726 | Slc38a10 | solute carrier family 38, member 10 | 2003.01 | 1574.25 | -0.35 | 7.56E-02 |  |
| ENSMUSG00000061171 | GSMG0022904 | Slc38a11 | solute carrier family 38, member 11 | 139.01 | 408.69 | 1.56 | 6.83E-14 |  |
| ENSMUSG00000022462 | GSMG0014445 | Slc38a2 | solute carrier family 38, member 2 | 35801.81 | 11728.93 | -1.61 | 9.30E-35 |  |
| ENSMUSG00000010064 | GSMG0041281 | Slc38a3 | solute carrier family 38, member 3 | 11390.56 | 11868.24 | 0.06 | 8.34E-01 | YES |
| ENSMUSG00000031170 | GSMG0041575 | Slc38a5 | solute carrier family 38, member 5 | 25444.55 | 14266.52 | -0.83 | 2.16E-06 |  |
| ENSMUSG00000044712 | GSMG0008178 | Slc38a6 | solute carrier family 38, member 6 | 188.64 | 173.58 | -0.12 | 7.83E-01 |  |
| ENSMUSG00000036534 | GSMG0038799 | Slc38a7 | solute carrier family 38, member 7 | 307.71 | 226.04 | -0.44 | 1.36E-01 |  |
| ENSMUSG00000047789 | GSMG0043708 | Slc38a9 | solute carrier family 38, member 9 | 402.24 | 466.84 | 0.22 | 4.52E-01 |  |
| ENSMUSG00000052310 | GSMG0024570 | Slc39a1 | solute carrier family 39 (zinc transporter), member 1 | 319.46 | 238.73 | -0.42 | 1.64E-01 |  |
| ENSMUSG00000025986 | GSMG0001550 | Slc39a10 | solute carrier family 39 (zinc transporter), member 10 | 4118.04 | 3938.09 | -0.06 | 8.47E-01 | YES |
| ENSMUSG00000041654 | GSMG0007594 | Slc39a11 | solute carrier family 39 (metal ion transporter), member 11 | 1131.22 | 617.95 | -0.87 | 1.04E-08 |  |
| ENSMUSG00000036949 | GSMG0020689 | Slc39a12 | solute carrier family 39 (zinc transporter), member 12 | 136.70 | 404.63 | 1.57 | 6.04E-12 |  |
| ENSMUSG00000002105 | GSMG0023165 | Slc39a13 | solute carrier family 39 (metal ion transporter), member 13 | 293.13 | 351.89 | 0.26 | 3.25E-01 |  |
| ENSMUSG00000046822 | GSMG0004354 | Slc39a3 | solute carrier family 39 (zinc transporter), member 3 | 1780.57 | 2170.19 | 0.29 | 2.15E-01 |  |
| ENSMUSG00000024270 | GSMG0018782 | Slc39a6 | solute carrier family 39 (metal ion transporter), member 6 | 1664.95 | 780.50 | -1.09 | 1.36E-13 |  |
| ENSMUSG00000053897 | GSMG0024979 | Slc39a8 | solute carrier family 39 (metal ion transporter), member 8 | 9492.75 | 5332.79 | -0.83 | 1.23E-07 | YES |
| ENSMUSG00000048833 | GSMG0008265 | Slc39a9 | solute carrier family 39 (zinc transporter), member 9 | 334.62 | 292.90 | -0.19 | 5.35E-01 |  |
| ENSMUSG00000010095 | GSMG0020062 | Slc3a2 | solute carrier family 3 (activators of dibasic and neutral amino acid transport), member 2 | 7707.96 | 5050.76 | -0.61 | 4.87E-05 |  |
| ENSMUSG00000025993 | GSMG0001548 | Slc40a1 | solute carrier family 40 (iron-regulated transporter), member 1 | 14991.82 | 14595.54 | -0.04 | 8.79E-01 | YES |
| ENSMUSG00000013275 | GSMG0000757 | Slc41a1 | solute carrier family 41, member 1 | 2635.24 | 2478.59 | -0.09 | 7.58E-01 |  |
| ENSMUSG00000034591 | GSMG0004394 | Slc41a2 | solute carrier family 41, member 2 | 104.13 | 82.87 | -0.34 | 4.97E-01 |  |
| ENSMUSG00000030089 | GSMG0032219 | Slc41a3 | solute carrier family 41, member 3 | 168.45 | 152.39 | -0.14 | 7.57E-01 |  |
| ENSMUSG00000038178 | GSMG0005530 | Slc43a2 | solute carrier family 43, member 2 | 1759.88 | 1121.59 | -0.65 | 2.33E-03 |  |
| ENSMUSG00000027074 | GSMG0021314 | Slc43a3 | solute carrier family 43, member 3 | 1328.43 | 617.18 | -1.10 | 1.52E-09 |  |
| ENSMUSG00000028412 | GSMG0026565 | Slc44a1 | solute carrier family 44, member 1 | 2425.27 | 3331.57 | 0.46 | 1.71E-02 |  |
| ENSMUSG00000057193 | GSMG0039217 | Slc44a2 | solute carrier family 44, member 2 | 1639.52 | 1455.03 | -0.17 | 4.43E-01 |  |
| ENSMUSG00000028360 | GSMG0025109 | Slc44a5 | solute carrier family 44, member 5 | 394.29 | 313.14 | -0.33 | 1.72E-01 |  |
| ENSMUSG00000039838 | GSMG0028687 | Slc45a1 | solute carrier family 45, member 1 | 416.12 | 400.25 | -0.06 | 9.00E-01 |  |
| ENSMUSG00000079020 | GSMG0014106 | Slc45a4 | solute carrier family 45, member 4 | 327.29 | 494.68 | 0.60 | 5.10E-02 |  |
| ENSMUSG00000020829 | GSMG0005597 | Slc46a1 | solute carrier family 46, member 1 | 187.10 | 141.97 | -0.39 | 2.12E-01 |  |
| ENSMUSG00000029650 | GSMG0031380 | Slc46a3 | solute carrier family 46, member 3 | 1556.03 | 1679.89 | 0.11 | 6.95E-01 |  |
| ENSMUSG00000081534 | GSMG0013576 | Slc48a1 | solute carrier family 48 (heme transporter), member 1 | 2859.58 | 4045.92 | 0.50 | 7.87E-04 |  |
| ENSMUSG00000006574 | GSMG0007445 | Slc4a1 | solute carrier family 4 (anion exchanger), member 1 | 343.91 | 155.06 | -1.16 | 3.30E-06 |  |
| ENSMUSG00000026904 | GSMG0021128 | Slc4a10 | solute carrier family 4, sodium bicarbonate cotransporter-like, member 10 | 908.71 | 946.86 | 0.06 | 8.47E-01 |  |
| ENSMUSG00000029141 | GSMG0029018 | Slc4a1ap | solute carrier family 4 (anion exchanger), member 1, adaptor protein | 378.09 | 386.12 | 0.03 | 9.33E-01 |  |
| ENSMUSG00000028962 | GSMG0028920 | Slc4a2 | solute carrier family 4 (anion exchanger), member 2 | 1202.18 | 1274.65 | 0.08 | 7.84E-01 |  |
| ENSMUSG00000006576 | GSMG0000452 | Slc4a3 | solute carrier family 4 (anion exchanger), member 3 | 4772.46 | 5378.22 | 0.17 | 6.20E-01 |  |
| ENSMUSG00000060961 | GSMG0029384 | Slc4a4 | solute carrier family 4 (anion exchanger), member 4 | 917.95 | 1021.79 | 0.15 | 6.51E-01 |  |
| ENSMUSG00000021733 | GSMG0011242 | Slc4a7 | solute carrier family 4, sodium bicarbonate cotransporter, member 7 | 991.41 | 661.48 | -0.58 | 4.86E-04 |  |
| ENSMUSG00000023032 | GSMG0013651 | Slc4a8 | solute carrier family 4 (anion exchanger), member 8 | 509.27 | 372.96 | -0.45 | 5.46E-02 |  |
| ENSMUSG00000027953 | GSMG0025647 | Slc50a1 | solute carrier family 50 (sugar transporter), member 1 | 1554.46 | 2332.98 | 0.59 | 9.89E-05 |  |
| ENSMUSG00000022560 | GSMG0013311 | Slc52a2 | solute carrier protein 52, member 2 | 2314.94 | 1866.67 | -0.31 | 8.35E-02 |  |
| ENSMUSG00000027463 | GSMG0021964 | Slc52a3 | solute carrier protein family 52, member 3 | 836.52 | 2015.60 | 1.27 | 8.48E-13 |  |
| ENSMUSG00000039680,ENSMUSG00000089774 | GSMG0015311 | Slc5a3 // Mrps6 | solute carrier family 5 (inositol transporters), member 3 // mitochondrial ribosomal protein S6 | 1210.11 | 983.85 | -0.30 | 1.82E-01 |  |
| ENSMUSG00000000792 | GSMG0038569 | Slc5a5 | solute carrier family 5 (sodium iodide symporter), member 5 | 452.90 | 813.63 | 0.84 | 1.88E-05 |  |
| ENSMUSG00000006641 | GSMG0043514 | Slc5a6 | solute carrier family 5 (sodium-dependent vitamin transporter), member 6 | 1938.56 | 2997.89 | 0.63 | 1.39E-04 |  |
| ENSMUSG00000030310 | GSMG0032346 | Slc6a1 | solute carrier family 6 (neurotransmitter transporter, GABA), member 1 | 1373.84 | 1252.35 | -0.13 | 7.32E-01 |  |
| ENSMUSG00000030307 | GSMG0032345 | Slc6a11 | solute carrier family 6 (neurotransmitter transporter, GABA), member 11 | 916.94 | 517.91 | -0.82 | 1.03E-03 |  |
| ENSMUSG00000030108 | GSMG0032429 | Slc6a13 | solute carrier family 6 (neurotransmitter transporter, GABA), member 13 | 1419.08 | 1967.23 | 0.47 | 5.60E-02 |  |
| ENSMUSG00000019894 | GSMG0003493 | Slc6a15 | solute carrier family 6 (neurotransmitter transporter), member 15 | 613.96 | 162.00 | -1.92 | 2.04E-17 |  |
| ENSMUSG00000027894 | GSMG0025900 | Slc6a17 | solute carrier family 6 (neurotransmitter transporter), member 17 | 106.01 | 119.05 | 0.17 | 7.47E-01 |  |
| ENSMUSG00000036814 | GSMG0041491 | Slc6a20a | solute carrier family 6 (neurotransmitter transporter), member 20A | 1978.47 | 4062.36 | 1.04 | 4.58E-09 |  |
| ENSMUSG00000030096 | GSMG0032228 | Slc6a6 | solute carrier family 6 (neurotransmitter transporter, taurine), member 6 | 15284.51 | 33069.37 | 1.11 | 1.08E-14 |  |
| ENSMUSG00000052026 | GSMG0019014 | Slc6a7 | solute carrier family 6 (neurotransmitter transporter, L-proline), member 7 | 891.70 | 1161.62 | 0.38 | 1.21E-01 |  |
| ENSMUSG00000019558 | GSMG0041842 | Slc6a8 | solute carrier family 6 (neurotransmitter transporter, creatine), member 8 | 399.13 | 642.31 | 0.69 | 3.21E-04 |  |
| ENSMUSG00000028542 | GSMG0026982 | Slc6a9 | solute carrier family 6 (neurotransmitter transporter, glycine), member 9 | 537.11 | 429.31 | -0.32 | 2.89E-01 |  |
| ENSMUSG00000041313 | GSMG0031384 | Slc7a1 | solute carrier family 7 (cationic amino acid transporter, y+ system), member 1 | 14438.85 | 8572.12 | -0.75 | 5.54E-08 | YES |
| ENSMUSG00000030495 | GSMG0034345 | Slc7a10 | solute carrier family 7 (cationic amino acid transporter, y+ system), member 10 | 522.11 | 767.18 | 0.56 | 8.96E-03 |  |
| ENSMUSG00000027737 | GSMG0025365 | Slc7a11 | solute carrier family 7 (cationic amino acid transporter, y+ system), member 11 | 94.51 | 174.54 | 0.89 | 7.11E-03 |  |
| ENSMUSG00000069072 | GSMG0025283 | Slc7a14 | solute carrier family 7 (cationic amino acid transporter, y+ system), member 14 | 272.17 | 212.70 | -0.35 | 2.52E-01 |  |
| ENSMUSG00000031596 | GSMG0037222 | Slc7a2 | solute carrier family 7 (cationic amino acid transporter, y+ system), member 2 | 261.59 | 195.65 | -0.42 | 1.40E-01 |  |
| ENSMUSG00000031297 | GSMG0042716 | Slc7a3 | solute carrier family 7 (cationic amino acid transporter, y+ system), member 3 | 924.66 | 932.12 | 0.01 | 9.73E-01 |  |
| ENSMUSG00000022756 | GSMG0015506 | Slc7a4 | solute carrier family 7 (cationic amino acid transporter, y+ system), member 4 | 979.02 | 1173.87 | 0.26 | 2.65E-01 |  |
| ENSMUSG00000040010 | GSMG0039010 | Slc7a5 | solute carrier family 7 (cationic amino acid transporter, y+ system), member 5 | 100133.82 | 75622.81 | -0.41 | 2.76E-02 | YES |
| ENSMUSG00000031904 | GSMG0037747 | Slc7a6 | solute carrier family 7 (cationic amino acid transporter, y+ system), member 6 | 520.20 | 520.50 | 0.00 | 9.98E-01 |  |
| ENSMUSG00000033106 | GSMG0038869 | Slc7a6os | solute carrier family 7, member 6 opposite strand | 535.54 | 485.26 | -0.14 | 5.84E-01 |  |
| ENSMUSG00000000958 | GSMG0012393 | Slc7a7 | solute carrier family 7 (cationic amino acid transporter, y+ system), member 7 | 557.64 | 209.45 | -1.41 | 1.73E-11 |  |
| ENSMUSG00000022180 | GSMG0012403 | Slc7a8 | solute carrier family 7 (cationic amino acid transporter, y+ system), member 8 | 3456.42 | 1481.37 | -1.22 | 2.10E-17 |  |
| ENSMUSG00000054640 | GSMG0017897 | Slc8a1 | solute carrier family 8 (sodium/calcium exchanger), member 1 | 672.42 | 418.43 | -0.68 | 9.50E-04 |  |
| ENSMUSG00000032754 | GSMG0029731 | Slc8b1 | solute carrier family 8 (sodium/lithium/calcium exchanger), member B1 | 329.52 | 412.10 | 0.32 | 1.99E-01 |  |
| ENSMUSG00000028854 | GSMG0027186 | Slc9a1 | solute carrier family 9 (sodium/hydrogen exchanger), member 1 | 1078.09 | 971.70 | -0.15 | 4.94E-01 |  |
| ENSMUSG00000026062 | GSMG0000186 | Slc9a2 | solute carrier family 9 (sodium/hydrogen exchanger), member 2 | 82.52 | 198.48 | 1.27 | 4.48E-05 |  |
| ENSMUSG00000020733 | GSMG0006095 | Slc9a3r1 | solute carrier family 9 (sodium/hydrogen exchanger), member 3 regulator 1 | 526.30 | 465.61 | -0.18 | 4.88E-01 |  |
| ENSMUSG00000002504 | GSMG0017229 | Slc9a3r2 | solute carrier family 9 (sodium/hydrogen exchanger), member 3 regulator 2 | 10671.52 | 17664.94 | 0.73 | 1.73E-07 |  |
| ENSMUSG00000014786 | GSMG0037719 | Slc9a5 | solute carrier family 9 (sodium/hydrogen exchanger), member 5 | 813.77 | 642.94 | -0.34 | 2.02E-01 |  |
| ENSMUSG00000060681 | GSMG0041765 | Slc9a6 | solute carrier family 9 (sodium/hydrogen exchanger), member 6 | 549.84 | 374.20 | -0.56 | 6.79E-03 |  |
| ENSMUSG00000037341 | GSMG0042390 | Slc9a7 | solute carrier family 9 (sodium/hydrogen exchanger), member 7 | 103.74 | 82.19 | -0.33 | 4.34E-01 |  |
| ENSMUSG00000039463 | GSMG0060441 | Slc9a8 | solute carrier family 9 (sodium/hydrogen exchanger), member 8 | 455.12 | 346.20 | -0.40 | 1.21E-01 |  |
| ENSMUSG00000031129 | GSMG0039920 | Slc9a9 | solute carrier family 9 (sodium/hydrogen exchanger), member 9 | 272.56 | 360.16 | 0.41 | 1.99E-01 |  |
| ENSMUSG00000030237 | GSMG0033846 | Slco1a4 | solute carrier organic anion transporter family, member 1a4 | 3566.88 | 18971.56 | 2.41 | 1.03E-62 | YES |
| ENSMUSG00000030235 | GSMG0032619 | Slco1c1 | solute carrier organic anion transporter family, member 1c1 | 49002.66 | 52079.32 | 0.09 | 7.52E-01 | YES |
| ENSMUSG00000032548 | GSMG0040006 | Slco2a1 | solute carrier organic anion transporter family, member 2a1 | 1396.59 | 757.99 | -0.88 | 2.01E-06 |  |
| ENSMUSG00000030737 | GSMG0036314 | Slco2b1 | solute carrier organic anion transporter family, member 2b1 | 5656.46 | 5573.07 | -0.02 | 9.43E-01 | YES |
| ENSMUSG00000025790 | GSMG0036124 | Slco3a1 | solute carrier organic anion transporter family, member 3a1 | 1575.73 | 1997.45 | 0.34 | 3.32E-02 |  |
| ENSMUSG00000038963 | GSMG0022297 | Slco4a1 | solute carrier organic anion transporter family, member 4a1 | 261.95 | 103.80 | -1.34 | 3.23E-08 |  |
| ENSMUSG00000025938 | GSMG0001318 | Slco5a1 | solute carrier organic anion transporter family, member 5A1 | 119.50 | 48.06 | -1.30 | 6.90E-05 |  |
| NSMUSG00000015290,ENSMUSG00000032806 | GSMG0042614 | Ubl4a // Gm44504 // Slc10a3 | ubiquitin-like 4A // predicted readthrough transcript (NMD candidate), 44504 // solute carrier family 10 (sodium/bile acid cotransporter family), member 3 | 367.31 | 318.20 | -0.21 | 4.32E-01 |  |
